# Partnering With Caregivers and Clinicians to Determine Research Priorities in Pediatric Migrant Health

**DOI:** 10.1001/jamanetworkopen.2026.26087

**Published:** 2026-07-29

**Authors:** Veronika Wiemker, Farah Kazi, Maria Marcolin, Ibrahim Alothman, Farat Ara, Michael Asonganyi, Leila Bianchi, Valentina Burzio, Afona Chernet, Zabihullah Khrosh, Shpresa Matmuja, Bezawit Sima, Mariia Teslenko, Julia Haag, Ann de Guchtenaere, Toto Gronlund, Susitha Wanigaratne, Astrid Guttmann, Santino Severoni, Olena Nyankovska, Alexandra Kruse, Siobhán Neville, Nicole Weydmann, Julia Brandenberger

**Affiliations:** 1 Section of Health Equity Studies and Migration, Department of General Practice and Health Services Research, Heidelberg University Hospital, Heidelberg, Germany; 2 Department of Neonatology, Center for Pediatric and Adolescent Medicine, Heidelberg University Hospital, Heidelberg, Germany; 3 Division of Pediatric Emergency Medicine, Department of Pediatrics, Inselspital, Bern University Hospital, University of Bern, Bern, Switzerland; 4 University College Hospitals London NHS Foundation Trust, London, United Kingdom; 5 Medical Faculty, Witten/Herdecke University, Witten, Germany; 6 Birmingham, United Kingdom; 7 Faculty of Social and Behavioral Sciences, University of Linköping, Linköping, Sweden; 8 Pediatric Infectious Disease Unit, Meyer Children’s Hospital IRCCS, Florence, Italy; 9 Department of Pediatrics, Maggiore della Carità University Hospital, Novara, Italy; 10 Swiss Tropical and Public Health Institute, Allschwil, Switzerland; 11 University of Basel, Basel, Switzerland; 12 Faculty of Medicine, Balkh University, Balkh, Afghanistan; 13 National Institute for Health, Migration and Poverty, Rome, Italy; 14 Faculty of Health Sciences, Oslo Metropolitan University, Oslo, Norway; 15 Pediatric Service, Wilanów Hospital, Warsaw, Poland; 16 Ukrainian Academy of Pediatric Specialties, Kyiv, Ukraine; 17 Faculty of Health, Medical and Life Science, Furtwangen University, Furtwangen, Germany; 18 Department of Pediatrics, Ghent University, Ghent, Belgium; 19 The European Academy of Pediatrics, Brussels, Belgium; 20 James Lind Alliance, National Institute for Health and Care Research, Southampton, United Kingdom; 21 Edwin S.H. Leong Centre for Healthy Children, University of Toronto and The Hospital for Sick Children, Toronto, Ontario, Canada; 22 ICES, Toronto, Ontario, Canada; 23 Dalla Lana School of Public Health, University of Toronto, Toronto, Ontario, Canada; 24 Department of Pediatrics, University of Toronto, Toronto, Ontario, Canada; 25 Division of Pediatric Medicine, The Hospital for Sick Children, Toronto, Ontario, Canada; 26 Health and Migration Programme, Division of Universal Health Coverage and Healthier Populations, Department of Health and Migration, World Health Organization, Geneva, Switzerland; 27 Danylo Halytsky Lviv National Medical University, Lviv, Ukraine; 28 Faculty of Health Sciences and Psychology, Collegium Medicum, University of Rzeszów, Rzeszów, Poland; 29 Department of Pediatrics and Adolescence, Copenhagen University Hospital–Hvidovre, Copenhagen, Denmark; 30 Department of Clinical Medicine, Faculty of Health and Medicine, University of Copenhagen, Copenhagen, Denmark; 31 School of Medicine, University of Limerick, Limerick, Ireland; 32 Department of Pediatrics, University Hospital Limerick, Limerick, Ireland

## Abstract

**Importance:**

Pediatric migrant health has been identified by the World Health Organization as a critical area for interdisciplinary research to improve care for children and adolescents with migration experience. Nevertheless, research agendas have rarely been shaped systematically by individuals with lived and professional experience, limiting relevance and impact.

**Objective:**

To identify and prioritize the most important unanswered research questions in pediatric migrant health in Europe through a structured, participatory priority-setting process.

**Design, Setting, and Participants:**

This multiphase survey study (April 2024 to June 2025), led by migrants and clinicians using the James Lind Alliance participatory priority-setting methodology, comprised 2 online consultations informed by Delphi procedures and a final in-person consensus workshop using a modified nominal group technique. Participants residing in multiple European countries included migrant caregivers, former migrant children and adolescents, health care workers in pediatric migrant health, and double experts with combined lived and professional experience. They were recruited via professional networks, community organizations, and open calls. The final in-person consensus workshop convened in Basel, Switzerland, in June 2025.

**Main Outcomes and Measures:**

The primary outcome was a ranked list of the top 10 unanswered research priorities in pediatric migrant health based on participant-generated questions and consensus methods.

**Results:**

In consultation 1, 256 participants (156 [61.0%] with lived migration experience; 25 countries of residence, 41 countries of origin; 115 aged <35 years [44.9%], 138 aged ≥35 years [53.9%]; 158 female [61.7%]) submitted 1589 questions and comments, which were consolidated into 53 unanswered summary questions after qualitative content analysis and evidence checking. In consultation 2, rankings from 576 participants (214 [37.2%] with lived migration experience; 31 countries of residence, 50 countries of origin; 193 aged ≤35 years [33.5%], 364 aged ≥35 years [63.2%]; 412 female [71.5%]) yielded a short list of 25 questions. During the final consensus workshop, participants selected the top 10 research priorities. The 3 highest ranked priorities focused on universal access to health care, the health impact of racism and discrimination, and barriers to accessing care. Remaining priorities addressed health effects of migration, social determinants of health, needs of at-risk groups (including unaccompanied or undocumented minors and children with medical complexities), professional language support, training of health care workers, and family involvement in care.

**Conclusions and Relevance:**

The research priorities identified in this survey study could provide a roadmap for future multidisciplinary and participatory research to improve health equity for pediatric migrants in Europe.

## Key Points

**Question:**

What are the most important unanswered research priorities to improve health care for children and adolescents with migration experience in Europe?

**Findings:**

In this survey study using a multiphase participatory process, more than 500 participants, including migrant caregivers, former migrant children, and clinicians, across 32 countries, identified their top 10 research priorities. These priorities addressed health care access, discrimination and racism, health effects of migration, social determinants of health, needs of at-risk populations (unaccompanied or undocumented minors, children with medical complexity), and family involvement in care.

**Meaning:**

These stakeholder-driven priorities provide a framework to guide future research, policy, and practice to improve pediatric migrant health in Europe.

## Teaser

This survey study uses a participatory priority-setting process to identify the most important unanswered research questions in pediatric migrant health among migrant and clinician stakeholders in Europe.

## Introduction

Migration is a defining global force of the 21st century, shaping demographic, social, and economic patterns worldwide. In 2020, the latest year for which age-disaggregated data are available, an estimated 281 million people—approximately 3.6% of the global population—lived outside their country of birth. This population included more than 36 million children and adolescents younger than 18 years,^1^ approximately 6.7 million of them in Europe.^2,3^

Alone or with their families, children migrate for a wide spectrum of motives, ranging from voluntary relocation for education, parental employment, or social reasons to forced displacement due to conflict, climate change, or persecution. While migration may result in a variety of specific health risks and resilience factors,^4^ health systems and research in Europe remain implicitly designed for nonmigrant, stable populations and fail to adequately address the specific health needs of migrating children.^5,6^ As the World Health Organization’s (WHO’s) 2023 Global Research Agenda on Health, Migration, and Displacement highlighted, pediatric aspects have received insufficient attention within prioritized research themes.^7^ This deficiency in research and system design has serious implications for the realization of migrant children’s rights specified in the United Nations Convention on the Rights of the Child: to be heard; safeguarded from harm, exploitation, and discrimination; and have their basic needs met through access to health care, education, and an adequate standard of living.^8,9^

For some children and adolescents, migration can be a traumatic experience, causing toxic stress and long-term health disadvantages and resulting in a need for medical and psychosocial care.^10^ Leaving familiar environments can disrupt critical periods of physical, cognitive, and social development, with significant long-term effects on well-being, educational attainment, and mental health.^11,12^ Potential negative effects of migration are aggravated as children and young people with migration experience face restricted access to high-quality health care and preventive services^4^ while navigating language, legal, and socioeconomic barriers; trauma-related challenges; or discrimination and racism.^4,13,14^ These factors not only undermine the well-being of migrant children but also limit their future opportunities, thus harming society as a whole.^15^ Evidence-based, tailored support services are needed to nurture children and adolescent’s resilience, enabling them to adapt to new environments, acquire multilingual skills, and navigate multiple cultural contexts.^16,17^

Current pediatric migrant health research has predominantly focused on individual risk factors or clinical outcomes, with insufficient attention to structural and institutional forces. Children’s health is shaped within multilayered caregiving systems, including families, schools, health services, and communities, yet fragmented responsibilities and limited evidence for cross-sectoral solutions^18^ hamper coordinated support. Similarly, the cumulative impacts of restrictive legal status, family separation, racism, and socioeconomic marginalization are often studied separately rather than as reinforcing mechanisms of inequality, limiting our understanding of how inequities are produced and sustained across migration contexts.^4,19^

Although research has increasingly acknowledged the value of involving migrants, caregivers, and clinicians in codesigning studies and setting research priorities,^20^ meaningful and fully empowered engagement remains rare. Migrant children, caregivers, and frontline health care or social service workers—the individuals directly affected—are still underrepresented in research, leaving crucial experiential knowledge untapped and creating both a methodological and ethical gap.^18^

In response to this unmet need, the Refugees and Migrants in Europe–Adolescent and Child Health (REACH) Network was established in 2023 as a strategic advisory group of the European Academy of Pediatrics.^21,22^ The network comprises pediatricians and other health care workers from multiple European countries and seeks to leverage the potential of international collaboration and participatory research approaches to improve pediatric migrant health.

The Migrant Child and Adolescent Health–Research in Europe (Mi-CARE) study was initiated as the REACH Network’s first project and aimed to identify and rank the most urgent unanswered research priorities in pediatric migrant health across Europe. Drawing on the perspectives of affected stakeholders and communities, the study integrates the views of migrant caregivers, former migrant children and adolescents, and professionals working in health and social care. The overarching objective is to support the creation of a research agenda that is relevant, equitable, and ethical and reflects the realities of frontline pediatric health workers and children and families with migration experience.

## Methods

### Study Design

**Figure 1.**
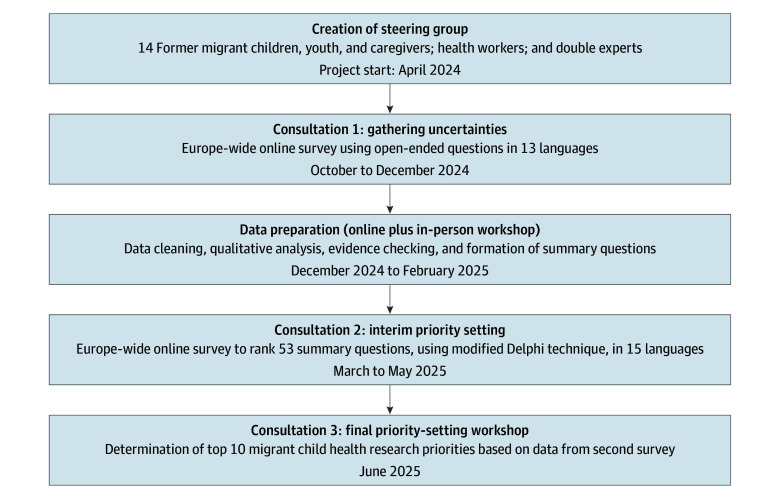
Flow Diagram of the 5 Project Phases of the Migrant Child and Adolescent Health–Research in Europe Priority Setting Partnership Survey Study

The Mi-CARE survey study followed the priority-setting partnership (PSP) methodology developed by the James Lind Alliance (JLA).^23^ Conducted between April 2024, and June 2025, this survey study comprised 5 consecutive phases (Figure 1; eMethods 1 in Supplement 1): (1) creation of the steering group; (2) consultation 1, European-wide stakeholder survey to identify unanswered research questions; (3) data preparation and synthesis; (4) consultation 2, European-wide stakeholder survey to rank research priorities; and (5) consultation 3, final consensus workshop. Stakeholders with personal and/or professional experience in pediatric migrant health guided all phases of the project, consistent with the principles of co-creation in participatory research.^24^

Ethical clearance was received by the Bern Ethics Committee (BASEC No. Req-2024-00587). Survey completion was voluntary, anonymous, and implied informed consent. The study followed the Reporting Guideline for Priority Setting of Health Research (REPRISE)^25^ and, where applicable, adhered to the American Association for Public Opinion Research Best Practices for Survey Research (AAPOR) reporting guideline.

### Steering Group

A steering group was established to anchor the PSP in lived experience. It included 4 former migrant children, adolescents, or migrant parents, 5 health care workers, and 5 individuals with double expertise (as a migrant and health care worker) (eMethods 1 and eTable 1 in Supplement 1). Members were recruited via the REACH Network and met monthly online, facilitated by a JLA advisor (T.G.) and PSP coordinators (V.W., F.K., and J.B.). The steering group guided the scope definition, survey development and dissemination, data interpretation, and knowledge translation and participated in in-person meetings during data preparation and the final workshop.

### Scope

This PSP focused on pediatric migrant health research in Europe, addressing the health-related needs of migrant children and young people aged 18 years or younger, including both refugee and nonrefugee minors. The aim was to identify research priorities applicable across diverse experiences and subgroups, assuming shared risk factors and health vulnerabilities across premigration, perimigration, and postmigration phases and developmental stages.^21^ General pediatric topics not specific to migration and country-system–specific questions were excluded. For all consultations, respondents self-identifying as migrant children, migrant parents or caregivers, or professionals working with migrant children in Europe to improve their health were included as relevant stakeholders. There were no formal exclusion criteria.

### Multilanguage Translation

**Figure 2.**
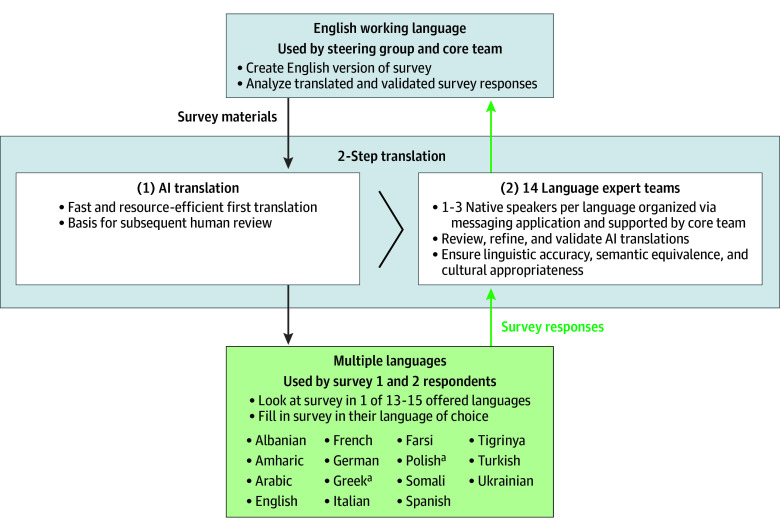
Schematic Diagram of the Hybrid Human Language Expert–Artificial Intelligence (AI) Translation Approach Used for Multilanguage Translation ^a^Survey 2 only.

For linguistic accessibility, a hybrid artificial intelligence–human translation approach was used. Survey materials and responses were translated to and from English using ChatGPT, version GPT-4o (OpenAI) and reviewed by teams of 1 to 3 native speakers (steering group members and authors’ personal contacts) coordinated by V.W. and F.K. Surveys and explanatory videos were translated into 12 languages (first survey) and 14 languages (second survey) (Figure 2).

### Consultation 1: Gathering Uncertainties

The first survey was developed in English, iteratively refined by the steering group, and piloted within the REACH Network to maximize accessibility and minimize suggestive prompting. Relevant stakeholders were invited to submit unanswered questions on pediatric migrant health from October 14 to December 31, 2024, via an online survey (SurveyMonkey)^26^ or on paper.

Survey links and print versions were disseminated through steering group members’ professional and personal networks, community partners, and social media using convenience and snowball sampling with multilingual materials (eMethods 2 in Supplement 1). The survey included 2 open-ended questions on health care challenges and potential improvements for migrant children and adolescents, supported by a short explanatory video and visual prompts (eMethods 3 in Supplement 1). Participants indicated their role (migrant caregiver or parent, unaccompanied or accompanied minor, health care worker, or double expert) and could provide demographic and migration information. No identifying data were collected, and no financial incentives were provided.

### Developing Summary Questions

Out-of-scope, unclear, or nonquestion survey responses were removed. The remaining submissions were analyzed using systematic content structuring analysis based on Kuckartz and Rädiker^27^ to identify summary topics, under which responses were grouped and condensed into summary questions. Although no formal weighting was applied, stakeholder group affiliation was tracked and considered throughout the iterative qualitative process to ensure balanced representation of inputs from migrant caregivers, health workers, and double experts. Oversight by the steering group, composed to reflect a range of relevant stakeholder characteristics across different countries and lived experiences, further ensured the inclusion of diverse perspectives. The summarizing process continued during monthly steering group meetings and an in-person workshop on December 5, 2024, with each steering group member responsible for coordinating question development for 1 or more topics to ensure accuracy, clarity, and accessibility.

### Evidence Checking

Summary questions were checked against evidence published in PubMed, Google Scholar, or the Cochrane Library by a team of physicians and researchers working in pediatric migrant health to determine whether they were unanswered (eMethods 4 in Supplement 1). Questions were defined as unanswered if evidence was absent, limited to single-country studies, or lacking systematic reviews or conclusive findings. Final decisions were agreed by consensus with steering group input.

### Consultation 2: Short-Listing Priorities

In the second online survey (March 20 to May 20, 2025) (eMethods 5 in Supplement 1), respondents were asked to select all summary questions they considered important from a randomly ordered list and to subsequently select up to 10 of these as priorities. Recruitment, target audience, and consent collection mirrored consultation 1. The data analysis included assigning 1 vote to each question ranked in a respondent’s top 10. Rankings were stratified by stakeholder role to highlight group-specific priorities. After reviewing for overlaps or missing themes, the steering group finalized a 25-question short list for consultation 3.

### Consultation 3: Final Priority Setting Workshop

The final workshop (eMethods 6 in Supplement 1) was convened in Basel, Switzerland, from June 4 to 8, 2025. All 14 steering group members were invited, of whom 6 agreed to participate. Additional participants were purposefully recruited via the REACH Network and the personal networks of steering group members to achieve a target of 15 to 20 participants. Selection aimed to ensure balanced representation across regions of origin and residence in Europe, migration experiences (including both refuge and other migration reasons), and stakeholder type (former migrant children, migrant caregivers, health care professionals, and double experts). Using a modified nominal group technique,^23^ JLA advisors (T.G. and 2 nonauthor collaborators) facilitated 3 small groups with balanced representation from all stakeholder groups. Each group ranked the 25 research priorities short-listed in consultation 2 and identified their top 10. Group rankings were combined using geometric means and presented in a plenary session for discussion and final agreement. Six international observers documented the process (V.W., F.K., S.W., A.G., N.W., and J.B.). Participation was voluntary, with travel, accommodation, childcare, and meals covered.

### Statistical Analysis

The data were analyzed using R, version 4.4.0 (R Foundation for Statistical Computing).

## Results

### Consultation 1: Gathering Uncertainties

**Figure 3.**
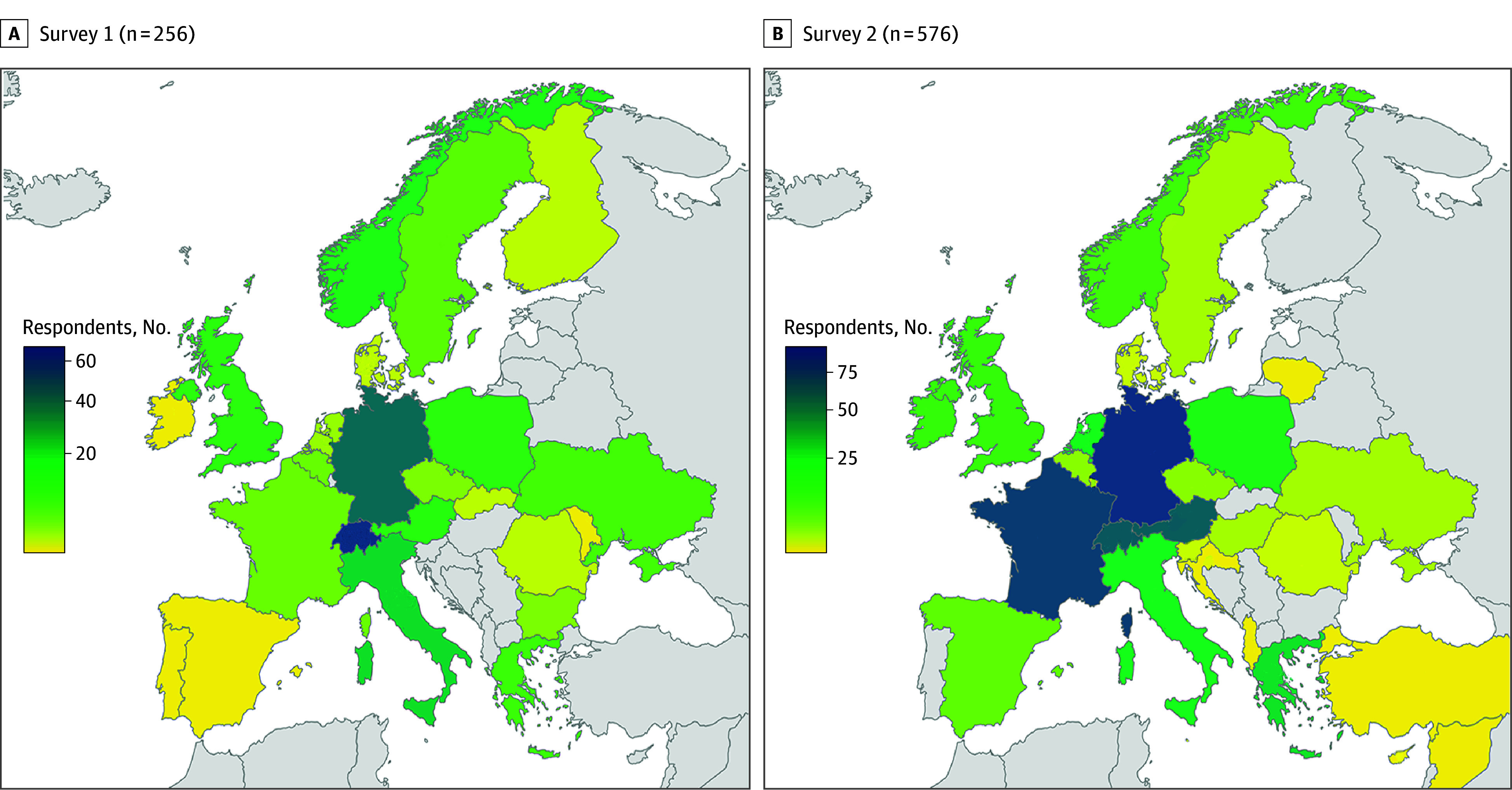
Map Showing Survey Respondents’ Current Countries of Residence Participants self-identified as migrants to Europe, which may have included individuals who were residing outside of Europe at the time of participation (eg, due to subsequent relocation or return migration). A response rate could not be calculated as the survey was disseminated via open, multichannel recruitment (including professional networks, community partners, and social media), and the number of individuals exposed to the invitation was unknown.

The first survey was completed by 256 respondents, including 156 individuals with lived migration experience (60.9%) and 133 clinicians (52.0%). Of these individuals, 33 (12.9%) had both perspectives (Table 1). Respondents (115 aged <35 years [44.9%], 138 aged ≥35 years [53.9%], 3 no response [1.2%]) were predominantly female (158 [61.7%] vs 94 male [36.7%] and 4 who preferred not to say or gave no response [1.6%]) and resided in 25 countries, mainly Switzerland (68 [26.6%]), Germany (47 [18.4%]), and Italy (26 [10.2%]) (Figure 3). Respondents with migration experience originated from 41 countries across all 6 WHO regions, predominantly the Eastern Mediterranean (65 [41.7%]), Europe (46 [29.5%]), and Africa (34 [21.8%]) compared with 5 from the Americas, Southeast Asia, and Western Pacific (2.0%) and 6 who did not answer (3.8%).

**Table 1.** Demographic Characteristics of Survey Respondents

Characteristic	Respondents, No. (%)	
	Survey 1 (n = 256)	Survey 2 (n = 576)
Role		
Migrant child or adolescent	55 (21.5)	49 (8.5)
Migrant parent or caregiver	68 (26.6)	94 (16.3)
Double expert	33 (12.9)	71 (12.3)
Health care worker	100 (39.1)	362 (62.8)
Sex		
Female	158 (61.7)	412 (71.5)
Male	94 (36.7)	138 (24.0)
Preferred not to say	2 (0.8)	5 (0.9)
Not answered	2 (0.8)	21 (3.6)
Age group, y		
<18	14 (5.5)	9 (1.6)
18-24	35 (13.7)	26 (4.5)
25-34	66 (25.8)	158 (27.4)
35-44	65 (25.4)	191 (33.2)
45-54	34 (13.3)	104 (18.1)
55-64	35 (13.7)	55 (9.5)
>65	4 (1.6)	14 (2.4)
Not answered	3 (1.2)	19 (3.3)
Region of residence		
Western Europe	141 (55.1)	348 (60.4)
Northern Europe	39 (15.2)	43 (7.5)
Southern Europe	37 (14.5)	73 (12.7)
Eastern Europe	33 (12.9)	34 (5.9)
Outside Europe^a^	2 (0.8)	6 (1.0)
Not answered	4 (1.6)	72 (12.5)
Region of origin (migrants only)		
No. of respondents	156	214
Europe	46 (29.5)	96 (44.9)
Eastern Mediterranean	65 (41.7)	45 (21.0)
Africa	34 (21.8)	24 (11.2)
Americas	3 (1.9)	9 (4.2)
Southeast Asia	1 (0.6)	3 (1.4)
Western Pacific	1 (0.6)	2 (0.9)
Not answered	6 (3.8)	35 (16.4)

^a^ Participants self-identified as migrants to Europe, which may have included individuals who had previously lived in Europe and engaged with European health care systems but who were residing outside of Europe at the time of participation (eg, due to subsequent relocation or return migration).

The most frequently chosen survey and video languages were German (63 respondents [24.6%]), English (47 respondents [18.4%]), and Ukrainian (28 respondents [10.9%]). Among migrant children and caregivers, Arabic (21 [17.1%]), Farsi (18 [14.6%]), and Ukrainian (18 [14.6%]) were the predominant languages (eTable 2 and eFigure 1 in Supplement 1). Respondents submitted 1589 questions and comments, of which 148 (9.3%) were out of scope. The remaining 1441 submissions were iteratively condensed by the steering group into 53 summary questions (eTable 3 in Supplement 1).

### Data Preparation and Evidence Checking

None of the 53 summary questions were identified as fully answered based on existing evidence. Thus, all questions progressed to the consultation 2 short-listing survey (eTable 3 in Supplement 1).

### Consultation 2: Short-Listing Priorities

The second survey was completed by 576 participants (193 aged ≤34 years [33.5%], 364 aged ≥35 years [63.2%], 19 no response [3.3%]; 412 female [71.5%], 138 male [24.0%], and 26 preferred not to say or no response [4.5%]). Of the respondents, 214 (37.2%) had lived experience, 433 (75.1%) were clinicians, and 71 (12.3%) had double expertise. Respondents resided in 31 countries, most frequently Germany (95 [16.5%]), France (84 [14.6%]), Switzerland (70 [12.2%]), and Austria (68 [11.8%]), and indicated 50 countries of origin (Europe, 96 [44.9%]; Eastern Mediterranean, 45 [21.0%]; Africa, 24 [11.2%]; Americas, Southeast Asia, or Western Pacific, 14 [6.5%]; no response, 35 [16.4%]) (Table 1). As no personal identifiers or pseudonyms were collected, it was not possible to track overlap between participants in the first and second consultation. The primarily chosen survey and video languages overall were English (278 [48.3%]), German (236 respondents [41.0%]), and French (157 respondents [27.3%]), while among 143 migrant children and caregivers, Ukrainian (30 respondents [21.0%]), English (27 respondents [18.9%]), and Arabic (18 respondents [12.6%]) were the predominant languages (eTable 2 in Supplement 1).

Stratified analyses generated priority rankings for participants with lived experience, health workers, and double experts (eTable 4 in Supplement 1). Across groups, consistently prioritized topics included racism and discrimination, language barriers, chronic and complex health needs, health impacts of the asylum process, and mental health support for stress, anxiety, and trauma. Participants with migration experience, including double experts, emphasized structural access barriers, social determinants of health, and the role of schools and community programs. Clinicians, again including double experts, prioritized mental health research, chronic and complex health needs, access for illegalized children, and health care worker training. The final short list of 25 questions (eTable 5 in Supplement 1) reflected both shared priorities and distinct stakeholder perspectives.

### Consultation 3: Final Priority Setting Workshop

The 21 workshop participants (16 female [76.2%] and 5 male [23.8%]) (eTable 6 in Supplement 1) included individuals with lived experience of migration (7 [33.3%]), nonmigrant health care workers (6 [28.6%]), and double experts (8 [38.1%]) representing 9 countries of current residence; migrants represented 11 countries of origin. Informed by the short-listing consultation and cross-cutting themes identified throughout the PSP, they reached consensus on the top 10 research priorities (Table 2; eTable 7 and eFigure 2 in Supplement 1).^28^ Priorities focused on access to health care; the health effects of discrimination and racism; physical and mental health consequences of migration; social determinants of health; the needs of at-risk groups, including unaccompanied minors, undocumented children, and children with medical complexities; health care worker training; and increasing family involvement in care.

**Table 2.** Top 10 Unanswered Research Priorities in Pediatric Migrant Health^a^

Rank	Question
1	What strategies or interventions can ensure health care services for all migrant children and teenagers (universal access)?
2	What impact do racism and discrimination have on the quality of care and (long-term) health of migrant children?
3	What are the main barriers preventing migrant children, including those without documentation or health insurance, from accessing health care?
4	In what ways does migration impact the physical and mental health of children?
5	How can migrant children with chronic illnesses, disabilities, and complex health needs get timely and equitable access to health care (including medications, treatments, and assistive devices)?
6	How do social factors in the arrival country, such as living conditions, education, and income, affect the health of migrant children?
7	What are the most effective training approaches to help health care workers understand the health effects of migration on children?
8	What are the specific health care needs and challenges faced by unaccompanied minor refugees?
9	What are the most effective ways to ensure cost-free and easily accessible professional language support for migrant children in health care institutions?
10	How can health care services involve parents and families more in supporting the health of migrant children?

^a^ A video presentation of the top 10 research priorities is also available.^28^

Universal health care access for migrant children emerged as the top priority, as—despite not being in any group’s top 5 in consultation 2—participants emphasized that it encompassed several highly ranked topics related to legal, linguistic, and social barriers to care. Research on discrimination and racism (ranked among the top 2 priorities by respondents with migration experience and double experts) ranked second overall, and language barrier research, which ranked highest among respondents with migration experience, was included as priority 9. Some widely discussed topics, including vaccination and preventive health programs, the role of schools and community settings, and cultural humility training and retraumatization prevention, were considered part of included broader priorities.

## Discussion

In this survey study across a 5-phase participatory priority-setting process involving more than 500 people with lived experience of migration and/or clinical expertise residing in 32 European countries, stakeholders identified substantial unmet evidence needs in pediatric migrant health, particularly with regard to access barriers, discrimination, language support, and mental health. While the short-listing consultation revealed differing emphases, including structural access barriers and social determinants of health as highlighted by migrants and clinical and mental health needs as prioritized by health workers, a strong convergence emerged around cross-cutting research themes of health equity for migrant children. Final consensus positioned universal access to health care as the overarching research priority, integrating the need for evidence on strategies to overcome legal, linguistic, social, and systemic barriers.

The top priority research on strategies, models, and interventions to achieve universal health access for migrant children and young people aligns closely with the WHO Global Action Plan on Promoting the Health of Refugees and Migrants^29^ and responds to previous calls to address the underrepresentation of children and adolescents in migration health research.^4,7^ The findings extend existing agendas by grounding the priorities in stakeholder experience, specifying concrete pediatric research gaps across the care continuum, and placing the child and their developmental context at the center.

At-risk groups described in WHO frameworks^4,7,29^ are specified for the pediatric context as undocumented and unaccompanied children, as well as those with chronic illnesses. Additional priorities highlight gaps in evidence on how migration, as well as racism and discrimination, interact with developmental stages to shape children’s physical and mental health and on how health care professionals could be effectively trained in these areas. Identified system-level gaps include the need for coordinated evidence on how social determinants of health, such as housing, education, and income, in host countries shape pediatric health trajectories and for structured approaches to migrant family involvement in care. A draft for an operational strategic framework visualizing the identified priorities for researchers and funders was developed during the consensus workshop and further elaborated by the REACH Network (eFigure 2 in Supplement 1).

Meaningful co-creation with people with lived migration experience requires effective strategies to overcome language barriers.^30,31^ Within this PSP, a hybrid translation approach was adopted, combining initial translations generated using language models with subsequent review and validation by stakeholder native speakers. This approach reduced costs and improved efficiency while ensuring accuracy, cultural appropriateness, and trust through human oversight.

The importance of family and caregiver involvement was reflected both in the final research agenda and in the study methodology, which actively sought caregiver participation, acknowledging their central role in supporting children’s health and well-being.^32^ Participants with both lived migration experience and professional health care expertise (double experts) contributed particularly rich perspectives. Given the substantial proportion of migrants working in European health care systems, the study highlights the potential of these double experts to build trust, bridge cultural gaps, and strengthen engagement with marginalized communities.

To leverage the long-term impact of the PSP, an in-depth top 10 research priorities booklet was developed during the consensus workshop and finalized through a writing partnership involving steering group members and workshop participants. The top 10 booklet will provide contextual information, interpretative guidance, and examples of concrete research questions for each priority, providing a practical implementation resource for researchers, clinicians, and advocates. The Mi-CARE project also supported sustainable European-level network building, including collaboration with initiatives such as the REACH Network and the establishment of a Mi-CARE advisory board composed of participants with lived migration experience to enable ongoing work beyond this study.

### Limitations

While this study implemented multiple strategies to strengthen the generalizability and relevance of the results (eg, integrating a wide range of perspectives through the steering group, surveys, and final workshop and continuous stakeholder involvement and leadership), important limitations remain. Convenience and snowball sampling may have introduced bias. Direct participation of currently migrating children was beyond the scope of this PSP. Accessibility challenges included limited survey languages and no financial compensation beyond travel support and may have contributed to overrepresentation of stakeholders from Western Europe and underrepresentation of stakeholder populations facing multiple, intersecting forms of marginalization. Subgroup-specific priorities and policy implications will require targeted follow-up studies due to the PSP’s broad thematic and geographic scope, along with the heterogeneity of child migrant populations.

## Conclusions

This participative, multiphase, survey study involving more than 500 participants—migrant caregivers, former migrant children, and clinicians from 32 countries—identified these stakeholders’ top research priorities for pediatric migrant health. The identified priorities address health care access, discrimination and racism, health effects of migration, social determinants of health, needs of at-risk populations, and family involvement in care. They offer an empirically grounded agenda to guide investment, policy development, and evidence generation across Europe.

## Notes

Supplement 1. **eMethods 1.** JLA Priority Setting Partnership Protocol for Mi-CARE, July 30, 2024, Version 1 **eMethods 2.** FAQ Mi-CARE Flier With Links to Introductory Video and Example Fliers in Different Languages **eMethods 3.** First Mi-CARE Consultation, October 14 to December 31, 2024 **eMethods 4.** Evidence Checking Framework, February 28 to March 10, 2025 **eMethods 5.** Second Mi-CARE Consultation, March 20 to May 20, 2025 **eMethods 6.** Basel Final PSP Workshop Program **eTable 1.** Key Definitions of Population Groups in the Study **eTable 2.** Language Choices in the Mi-CARE Consultations **eTable 3.** List of the 53 Summary Questions **eTable 4.** Ranking Table by Respondent Group **eTable 5.** Top 25 Research Priorities on Pediatric Migrant Health in Europe, Based on the First and Second Mi-CARE Consultations (Random Order of Presentation) **eTable 6.** Mi-CARE Final Workshop Participants **eTable 7.** Mi-CARE Final Workshop: Combined Group’s Ranking of Top 25 Priorities (Arithmetic and Geometric Mean) **eFigure 1.** Language Distributions in the First and Second Mi-CARE Consultations by Respondents’ Role **eFigure 2.** Strategic Research Framework of Identified Priorities

Supplement 2.
Nonauthor Collaborators

Supplement 3.
Data Sharing Statement
