# Supplementary material for: Partnering With Caregivers and Clinicians to Determine Research Priorities in Pediatric Migrant Health
Source: JAMA Netw Open. 2026 Jul 29;9(7):e2626087. doi: 10.1001/jamanetworkopen.2026.26087 (PMC13421195; doi:10.1001/jamanetworkopen.2026.26087)
Supplement: Supplement 1. — eMethods 1. JLA Priority Setting Partnership Protocol for Mi-CARE, July 30, 2024, Version 1 eMethods 2. FAQ Mi-CARE Flier With Links to Introductory Video and Example Fliers in Different Languages eMethods 3. First Mi-CARE Consultation, October 14 to December 31, 2024 eMethods 4. Evidence Checking Framework, February 28 to March 10, 2025 eMethods 5. Second Mi-CARE Consultation, March 20 to May 20, 2025 eMethods 6. Basel Final PSP Workshop Program eTable 1. Key Definitions of Population Groups in the Study eTable 2. Language Choices in the Mi-CARE Consultations eTable 3. List of the 53 Summary Questions eTable 4. Ranking Table by Respondent Group eTable 5. Top 25 Research Priorities on Pediatric Migrant Health in Europe, Based on the First and Second Mi-CARE Consultations (Random Order of Presentation) eTable 6. Mi-CARE Final Workshop Participants eTable 7. Mi-CARE Final Workshop: Combined Group’s Ranking of Top 25 Priorities (Arithmetic and Geometric Mean) eFigure 1. Language Distributions in the First and Second Mi-CARE Consultations by Respondents’ Role eFigure 2. Strategic Research Framework of Identified Priorities [file jamanetwopen-e2626087-s001.pdf]

Supplemental Online Content

Wiemker V, Kazi F, Marcolin M, et al. Migrant caregiver and chlinician partnering to determine research priorities in pediatric migrant health. *JAMA Netw Open*. 2026;9(7):e2626087. doi:10.1001/jamanetworkopen.2026.26087

eMethods 1. JLA Priority Setting Partnership Protocol for Mi-CARE, July 30, 2024, Version 1

eMethods 2. FAQ Mi-CARE Flier With Links to Introductory Video and Example Fliers in Different Languages

eMethods 3. First Mi-CARE Consultation, October 14 to December 31, 2024

eMethods 4. Evidence Checking Framework, February 28 to March 10, 2025

eMethods 5. Second Mi-CARE Consultation, March 20 to May 20, 2025

eMethods 6. Basel Final PSP Workshop Program

eTable 1. Key Definitions of Population Groups in the Study

eTable 2. Language Choices in the Mi-CARE Consultations

eTable 3. List of the 53 Summary Questions

eTable 4. Ranking Table by Respondent Group

eTable 5. Top 25 Research Priorities on Pediatric Migrant Health in Europe, Based on the First and Second Mi-CARE Consultations (Random Order of Presentation)

eTable 6. Mi-CARE Final Workshop Participants

eTable 7. Mi-CARE Final Workshop: Combined Group's Ranking of Top 25 Priorities (Arithmetic and Geometric Mean)

eFigure 1. Language Distributions in the First and Second Mi-CARE Consultations by Respondents' Role

eFigure 2. Strategic Research Framework of Identified Priorities

This supplemental material has been provided by the authors to give readers additional information about their work.

© 2026 Wiemker V et al. *JAMA Network Open*.

eMethods 1. JLA Priority Setting Partnership Protocol for Mi-CARE, July 30, 2024, Version 1

Mi-CARE
MIgrant Child & Adolescent health - Research in Europe

Priority Setting Partnership
PROTOCOL 2024-07-30 Version 1

1. Purpose of the PSP and background

The purpose of this protocol is to clearly set out the aims, objectives and commitments of the **Paediatric Migrant Health in Europe** Priority Setting Partnership (PSP) in line with James Lind Alliance (JLA) principles.

The principles of the JLA are:

- **Transparency**: openness of process, including an audit trail of original submitted uncertainties, to final prioritised list
- **Inclusiveness**: balanced inclusion of the perspectives of people with lived experience, caregivers and health and care professionals
- **Evidence base**: ensuring candidate questions for prioritisation are formally verified as unanswered.
- **Exclusion of researchers in prioritisation** (they may be involved and helpful in all other aspects of the process)
- **Exclusion of groups/organisations that have significant competing or commercial interests**, for example pharmaceutical companies.

The aim of a PSP is to help ensure that those who fund health research are aware of what really matters to people with lived experience, caregivers and health and care professionals who look after them. PSPs bring these people together to identify and prioritise the unanswered questions that they agree are the most important for research to address in their topic area. This provides a valuable perspective to the research agenda, which may differ from the research priorities of industry, academics and governments.

The Protocol is a JLA requirement and will be published on the PSP's page of the JLA website. The Steering Group will review the Protocol regularly and any updated version will be sent to the JLA.

The JLA is a non-profit making initiative, established in 2004. The National Institute for Health and Care Research (NIHR – www.nihr.ac.uk) coordinates the infrastructure of the JLA to oversee the processes for PSPs, based at the NIHR Coordinating Centre (NIHRCC), University of Southampton.

Migration is a driver and enabler of sustainable development, and migrant health an important part of public health. Yet many health systems continue to be tailored exclusively to the non-migrant population. This leads to important health disparities for migrants and refugees, especially children and adolescents.

In this PSP, different groups of people directly affected by the (lack of) research in paediatric migrant health join their perspectives to prioritise research questions that need to be addressed.

In 2023, the Refugees and Migrants in Europe – Adolescent and Child Health (REACH) network became a Special Advisory Group of the European Academy of Paediatrics (EAP; https://www.eapaediatrics.eu/advisory-groups/reach/). It is composed of approximately 20 healthcare professionals actively involved in paediatric migrant healthcare in various countries of the European Region (e.g. Italy, Denmark, Germany, Switzerland, Netherlands, Poland, Great Britain, France). They are committed to the goal of providing equitable, excellent healthcare to all children and adolescents – regardless of their origin. Some REACH group members are 'double experts'; meaning that they have both lived experience of migration as well as experience working in the healthcare sector. In this PSP, the perspectives of the REACH health workers are combined with those of

© 2026 Wiemker V et al. *JAMA Network Open*.

parents/carers of children with migration experience using the host countries' healthcare systems and perspectives of people who migrated when they were still children/adolescents themselves.

The Mi-CARE PSP is supported by a networking grant of the Academy of Medical Sciences (UK) and is intended to be the starting point for future collaboration projects, such as larger research initiatives focusing on some of the identified research priorities or uncertainties.

2. Objectives and scope of the PSP

The objectives of the Mi-CARE PSP are to:

- work with people with lived experience, caregivers as well as health and care professionals to identify unanswered questions about the **health and optimal delivery of healthcare to children and adolescents with migration experience in Europe (including the whole spectrum from forced to voluntary migration)**
- to agree by consensus a prioritised list of those unanswered questions, for research
- to publicise the results of the PSP and process
- to take the results to research commissioning bodies to be considered for funding.

The scope of the PSP is defined as:

- Any aspect of the medical prevention, diagnosis, treatment, management and care of children and adolescents with migration experience in Europe
- Questions that may inform the optimal design of inclusive, effective, and resilient healthcare systems will be particularly focused upon
- We want to include a diversity of perspectives regarding the following aspects:
    - care levels: e.g. arrival centre, consultancy for general population (primary care), hospital (secondary, tertiary care)
    - regions of healthcare delivery: e.g. Eastern Europe; arrival countries (such as Italy, Greece)
    - medical fields within paediatrics: e.g. mental health, emergency care, management of chronic diseases
    - paediatric age groups: e.g. babies, toddlers, children, adolescents
    - migration routes: e.g. travel by foot, boat, plane
    - regions of patients' childhood: e.g. Syria, Ukraine, Nigeria, different European countries
    - sociodemographic privilege of patients in host country: e.g. "highly skilled" working migrants, forced vs. voluntary migration
    - vulnerabilities of patients: e.g. marginalised sexual orientation and gender identities

We recognise that this is a very broad remit. While we conceive this as a strength that may allow to find common themes that matter to a very large group of people that still often remains unheard, we also recognize that decisions may need to be made to further focus the scope of the PSP depending on the questions remaining after categorisation.

The PSP will exclude from its scope questions about:

- Healthcare of adults with migration experience
- Healthcare of children/adolescents residing outside of Europe
- Health uncertainties pertaining to child and adolescent health in general, that is, which affect children and adolescents with and without migration history equally

The Steering Group is responsible for discussing the implications of the scope on all of the stages of the process.

3. The Steering Group

The Steering Group includes membership of people with lived experience and caregivers and health and care professionals, as individuals or representatives from a relevant group.

People with lived experience, caregivers:

Michael Asonganyi, *filmmaker / producer / actor, residing in Norway, from Cameroon*
Farat Ara, *university teacher, residing in the United Kingdom, from Pakistan*
Vian Ronny, *teacher in public school, residing in Germany, from Syria*
Shpresa Matmuja, *language interpreter at the National Institute for Migration and Poverty, residing in Italy, from Albania*
Bezawit Sima, *guest researcher in Oslo, PhD in Health Sciences from the University of Oslo, residing in Norway, from Ethiopia*

Double Experts (people with lived experience who have a professional link to the healthcare sector):

Mariia Teslenko, *Paediatrician at in-patient hospital, doctor of philosophy, residing in Poland, from Ukraine*
Afona Chernet, *academic researcher at the Swiss Tropical and Public Health Institute, University of Basel, residing in Switzerland, from Eritrea*
Zabihullah Khrosh, *Doctor at medical faculty in Afghanistan, residing in Italy, from Afghanistan*
Ibrahim Alothman, *Medical student & nurse, residing in Germany, from Syria*

Healthcare professionals:

Leila Bianchi, *Paediatrician at the Meyer Children's Hospital in Florence, Italy*
Valentina Burzio, *Paediatrician at Azienda Ospedaliera Universitario, SC di Pediatria, Ospedale Maggiore della Carità di Novara, Novara, Italy*
Julia Brandenberger, *Paediatrician in paediatric emergency medicine at the Kinderklinik Universitätsspital Bern, Switzerland*
Farah Kazi, *Masterstudent of Medicine, University of Bern, Bern, Switzerland*
Veronika Wiemker, *Paediatrician in training, Heidelberg, Germany*

Supporters of the Steering Group:

PSP Lead(s): **Julia Brandenberger, Kinderklinik Universitätsspital Bern, Switzerland**
Project coordinator: **Farah Kazi, Universität Bern, Switzerland / Veronika Wiemker, Universitätsklinikum Heidelberg, Germany**
James Lind Alliance Adviser and Chair of the Steering Group: **Toto Gronlund, JLA**
Information specialist: **Nicole Weydmann, Hochschule Furtwangen, Germany**

The separate Terms of Reference outline the time and expertise that they are asked to contribute to each stage of the process, with input and advice from the JLA.

4. Partners

Organisations and individuals will be invited to be involved with the PSP as partners. Partners are organisations or groups who will commit to supporting the PSP, promoting the process and encouraging their represented groups or members to participate. Organisations which can reach and advocate for these groups will be invited to become involved in the PSP. Partners can represent the following groups:

- **children and adolescents with migration experience in Europe** (may be adults by now)
- caregivers of **children and adolescents with migration experience in Europe**
- health and social care professionals - with experience of **healthcare delivery to children and adolescents with migration experience in Europe**

Exclusion criteria
Some organisations may be judged by the JLA or the Steering Group to have conflicts of interest. These may be perceived to potentially cause unacceptable bias as a member of the Steering Group. As this is likely to affect the ultimate findings of the PSP, those organisations will not be invited to participate. It is possible, however, that interested parties may participate in a purely observational capacity when the Steering Group considers it may be helpful.

Partners:

© 2026 Wiemker V et al. *JAMA Network Open*.

Refugees and Migrants in Europe – Adolescent and Child Health (REACH) group, official Strategic Advisory Group of the **EAP (European Academy of Paediatrics**, Paediatric section of UEMS); see https://www.eapaediatrics.eu/advisory-groups/reach/

5. The methods the PSP will use

This section describes a schedule of proposed steps through which the PSP aims to meet its objectives. The process is iterative and dependent on the active participation and contribution of different groups. The methods used in any step will be agreed through consultation between the Steering Group members, guided by the PSP's aims and objectives. More details of the method are in the Guidebook section of the JLA website at www.jla.nihr.ac.uk where examples of the work of other JLA PSPs can be seen.

Step 1a: Forming the Steering Group and identification and invitation of potential partners

The Steering Group and potential partner organisations will be identified through a process of peer knowledge and consultation. Potential partners will be contacted and informed of the establishment and aims of the PSP. The Steering Group developed and agreed this protocol.

Step 1b: Awareness raising

PSPs will need to raise awareness of their proposed activity among their patient, carer and health and care professional communities, in order to secure support and participation. Depending on budget, this may be done by a face-to-face meeting, or there may be other ways in which the process can be launched, e.g. via social media. It may be carried out as part of steps 1 and/or 3. The Steering Group should advise on when to do this. Awareness raising has several key objectives:

- to present the proposed plan for the PSP
- to generate support for the process
- to encourage participation in the process
- to initiate discussion, answer questions and address concerns.

Step 2: Gathering unanswered questions

The PSP will carry out a consultation to gather unanswered questions from people with lived experience, caregivers and health and care professionals. A period of 3 months **(October - December 2024)** will be given to complete this exercise (which may be revised by the Steering Group if required).

The PSP recognises that the following groups/circumstances may require additional consideration:

A major challenge of this PSP is the **immense diversity within migrant and refugee populations**, as well as the **variability in healthcare settings across the European region**. This diversity spans a wide range of cultural and socioeconomic backgrounds, healthcare needs and available resources, and migration experiences. Additionally, the dynamic nature of migration patterns and demographics leads to evolving healthcare needs over time. The significant **variation of European healthcare systems** adds another layer of complexity.

These circumstances will likely be reflected in the submitted uncertainties, which we expect to cover a broad spectrum of health issues, as well as in the individual experiences of the Steering Group members. It will therefore be essential to clearly communicate the scope, focus and objectives of the PSP to survey respondents. The Steering Group must also allocate sufficient time to discuss and refine emerging overarching themes in order to distil clear summary questions from the submitted uncertainties.

Additional challenges arise in **reaching the most vulnerable populations, whose needs and perspectives are of particular interest.** Many migrants, particularly refugees, face unstable living conditions, which can hinder their participation in research initiatives. **Language barriers** between Steering Group Members and some groups of potential survey respondents further complicate this issue. There may also be a level of **distrust** among some migrants towards institutions and authorities **due to past experiences of discrimination or racism**.

These factors make trustful collaborations with migrant-led organisations and the use of personal connections between healthcare and social care professionals and patients / caregivers essential. We also aim to tailor specific

© 2026 Wiemker V et al. *JAMA Network Open*.

survey distribution approaches to different national contexts, use multilingual resources and explore the use of several surveys with differing levels of language complexity, videos/graphic illustration and translation options to mitigate language-barriers.

Within the survey we may include some questions to better understand the self-identified perspective of the respondent (e.g. “What country are you from?”, “What country are you seeking healthcare in?”, “What gender do you identify with?”, “What area of care can you provide insights on?”). This will enable us to develop targeted recruitment strategies for people with characteristics that are initially underrepresented.

The Steering Group will use the following methods to reach the target groups:

- As in all PSPs, the **Steering Group members** play a critical role in fostering trustful collaboration between patients/carers and clinicians. Individuals with lived experiences, in particular, will provide guidance on language, design, and inclusive engagement strategies during the survey preparation phase. They will also use their personal networks to broaden access to the PSP's online survey and publicise the results within their communities and networks.

- Healthcare professionals from the **REACH group**, may they be Steering Group members or not, will use various methods, such as **posters and direct communication** and to inform patients about the PSP in their clinics and practices and **engage medical colleagues in their local networks** to do the same. In some cases, language barriers may be addressed by Steering Group healthcare professionals who offer assistance with completing the online form in their practives. Additionally, the feasibility of providing multilingual inout options, which could then be translated by Steering Group members, will be explored.

- Whenever possible, **communication channels and presentation opportunities within the European Academy of Paediatrics (EAP)** will be used to promote participation in the planned PSP. To enhance representation of particularly marginalised groups, such as undocumented migrant communities or refugees in short-term accommodations that do not yet have access to standard care structures within the national health care system, the PSP aims to engage with migrant-led organisations in various European countries. These organisations will be asked to spread information about the PSP through their internal communication channels.

- **Collaboration with migrant-led organisations** is essential.

Existing sources of unanswered questions may also be searched:

- research recommendations in clinical guidelines; e.g., by WHO, EU, national governments/organizations
- research recommendations in systematic reviews (identified via Pubmed, Google Scholar etc.) ?
- protocols for systematic reviews being prepared and registers of ongoing research (identified via PROSPERO etc.)

Step 3: Refining questions and unanswered questions

As this is a consultation exercise, and most of the respondents are not researchers, responses are often not formed as neat questions, but may be statements or narratives.

These **raw submissions will be categorised and refined** by all Steering Group members, in particular **Farah Kazi and Veronika Wiemker,** supervised by **Nicole Weydmann** and potentially supported by assistance researchers from Nicole Weydmann's team into summary questions which are clear, addressable by research, and understandable to all. Similar or duplicate questions will be grouped where appropriate. Out-of-scope and ‘answered’ submissions will be compiled separately. The Steering Group will have engagement and oversight of this process to ensure that the raw data is being interpreted appropriately and that the summary questions are being worded in a way that is understandable to all audiences. The JLA Adviser will observe to ensure accountability and transparency.

This will result in a long list of in-scope summary questions. These are not research questions and to try and word them as such may make them too technical for a non-research audience. They will be framed as researchable questions that capture the themes and topics that people have suggested.

Step 4: Evidence checking

The summary questions will then be checked against evidence to determine whether they have already been answered by research. This will be done by members of the Steering Group potentially supported by assistance researchers from Nicole Weydmann's team. The PSP will complete the JLA Question Verification Form, to clearly describe the process used to verify the questions as unanswered by research. This must be done before starting prioritisation. The Question Verification Form asks for details of the types and sources of evidence that are used. The Question Verification Form should be published on the JLA website to enable researchers and other stakeholders to understand how the PSP has decided that its questions are unanswered, and any limitations of this.

Questions that are not adequately addressed by previous research will be collated and recorded on a standard JLA template. This will show the checking undertaken to make sure that the unanswered questions have not already been answered. The data should be submitted to the JLA for publication on its website on completion of the priority setting exercise, taking into account any changes made at the final workshop, in order to ensure that PSP results are publicly available.

The Steering Group will also consider how it will deal with submitted questions that have been answered, and submissions that are out of scope.

Step 5 & 6: Prioritisation – interim and final stages

The aim of the final stage of the priority setting process is to **prioritise through consensus the identified unanswered questions about Paediatric Migrant Health in Europ**e. This will involve input from people with lived experience, caregivers and health and care professionals. The JLA encourages PSPs to involve as wide a range of people as possible, including those who did and did not contribute to the first consultation. There are usually two stages of prioritisation.

1. **Shortlisting** is the stage where the long list of questions is reduced to a shorter list that can be taken to the final priority setting workshop. This is aimed at a wide audience, and is done using similar methods to the first consultation. With the JLA's guidance, the Steering Group will agree the method and consider how best to reach and engage people with lived experience, caregivers and health and care professionals in the process. The most highly ranked questions (around 20-25) will be taken to a final priority setting workshop. The results of the shortlisting are analysed by stakeholder group, as participation is rarely in equal numbers across these groups. Where the shortlisting does not produce a clear ranking or cut off point, the Steering Group will decide which questions are taken forwards to the final prioritisation.

2. The **final priority setting stage** is a one-day workshop facilitated by the JLA. With guidance from the JLA and input from the Steering Group, up to 30 people with lived experience, caregivers and health and care professionals will be recruited to participate in a day of discussion and ranking, to determine the top 10 questions for research. All participants will declare their interests. The Steering Group will advise on any adaptations needed to ensure that the process is inclusive and accessible.

Step 7. Dissemination of results

The Steering Group will identify audiences with which it wants to engage when disseminating the results of the priority setting process, such as researchers, funders and the patient and clinical communities. They will need to determine how best to communicate the results and who will take responsibility for this. Formats may include academic papers, lay reports, infographics, conference presentations and videos for social media.

It should be noted that the priorities are not worded as research questions. The Steering Group should discuss how they will work with researchers and funders to establish how to address the priorities and to work out what the research questions are that will address the issues that people have prioritised. The dissemination of the results of the PSP will be led by **Julia Brandenberger**.

The JLA encourages PSPs to report back about any activities that have come about because of the PSP, including funded research. Please send any details to jla@soton.ac.uk.

6. Agreement of the Steering Group

The **Paediatric Migrant Health in Europe** PSP Steering Group agreed the content and direction of this Protocol on July 30, 2024.

© 2026 Wiemker V et al. *JAMA Network Open*.

eMethods 2. FAQ Mi-CARE Flier With Links to Introductory Video and Example Fliers in Different Languages

Mi-CARE
MIgrant Child & Adolescent health – Research in Europe

Priority Setting Partnership
2024/2025

What is the Mi-CARE project about?

Children, adolescents, and families with migration experiences face unique and often daunting challenges when navigating the healthcare systems in Europe. From specific healthcare needs resulting from their migration journey, to language barriers and unfamiliar healthcare processes, they are often at a disadvantage when it comes to receiving the care they need. **Research addressing these challenges is essential** to ensure that every child, regardless of their background, receives the best possible healthcare.

The **MIgrant Child & Adolescent health – Research in Europe Priority Setting Partnership (Mi-CARE PSP)** is conducted in cooperation with the James Lind Alliance (JLA – https://www.jla.nihr.ac.uk/), a non-profit making initiative that brings together patients, carers and clinicians in JLA Priority Setting Partnerships (PSPs). JLA PSPs identify and prioritise unanswered questions in a specific area that they agree are the most important, so that researchers and funders are aware of the issues that matter most to the people.

The Mi-CARE project enables children, adolescents, and families with migration experience, and the healthcare professionals who work with them, to identify and prioritise their most important unanswered research questions in paediatric migrant health in Europe.

What do we do?

Following the JLA methodology, the Mi-CARE project has several steps:

Step 1 – Gathering unanswered questions:
A Europe-wide online survey is used to collect unanswered questions from people with lived experience of migration, caregivers, and healthcare professionals. We use multilingual resources, including videos and illustrations, to reduce language barriers and ensure broad participation. Collaborations with migrant-led organizations and personal connections between healthcare professionals, social workers, and patients help us reach migrant families, including those in vulnerable positions. **You can learn more about this step by watching the Mi-CARE survey introduction video**: https://youtu.be/OeppYnBZOHc

> In this step, we need your help! – see section *How can you support us?*

Refining and summarizing questions:
The survey respondents will not be researchers, so we do not expect their responses to be presented as clear research questions. We expect they will respond with concerns, ideas, as well as questions. Similar or duplicate questions will be grouped together to form researchable summary questions. Out-of-scope or already answered submissions will be compiled separately. The summary questions will then be checked to see if they have already been answered by existing research.

Step 2 – Prioritising researchable questions:
In this stage, the remaining summary questions about paediatric migrant health in Europe are prioritized through a consensus process, which involves two steps:

- **Shortlisting**: A second Europe-wide survey among people with lived experience, caregivers, and professionals will ask people to select their top priorities. The results of this shortlisting will be used to

© 2026 Wiemker V et al. *JAMA Network Open*.

identify the most important priorities (around 20-25 questions) to be taken to a final priority setting consensus workshop.

- **Final prioritization**: a one-day workshop facilitated by the JLA will bring together about 30 participants (people with lived experience, caregivers, and healthcare professionals) to rank and **determine the top 10 priorities.**

The Steering Group will oversee these two steps and advise on any adaptations needed to ensure that the process is inclusive and accessible for all.

How can you support us?

We are actively seeking individuals, organizations, and groups to support and promote the Mi-CARE project. Here is how you can help:

1. **Share your experiences and participate in the first Mi-CARE survey if you…**

- migrated to Europe as a child or teenager
- are a parent or carer who migrated with children or had children after arriving
- are a healthcare professional (doctor, nurse, social worker, psychologist, etc.) working with migrant children and teenagers

Take the survey here (available in many languages): https://www.research.net/r/XPF5XKH

2. **Spread the word: Share the survey with others in your networks or show the introductions video to a migrant parent / caregiver and help them complete the survey.**

We want to include a diversity of perspectives regarding the following aspects:

- Levels of care: e.g. arrival centre, consultancy for general population (primary care), hospital (secondary, tertiary care)
- Regions of healthcare delivery: e.g. Eastern Europe; arrival countries (such as Italy, Greece)
- Medical fields within paediatrics: e.g. mental health, emergency care, management of chronic diseases
- Paediatric age groups: e.g. babies, toddlers, children, adolescents
- Regions of patients' childhood: e.g. Syria, Ukraine, different African and European countries
- Sociodemographic privilege of patients in host country: e.g. "highly skilled" working migrants, forced vs. voluntary migration
- Vulnerabilities of patients: e.g. marginalised sexual orientation and gender identities

Replies from those hardest to reach are most precious to the project.

3. **We welcome organizations that can reach and advocate for these groups to join the Mi-CARE project. Please reach out to us if you'd like to get involved!**

What will happen with the project results?

The Mi-CARE PSP enables people with lived experience and front line healthcare professionals to agree their Top 10 list of the most important unanswered research questions in the field of paediatric migrant health in Europe. These priorities will serve as a starting point for future (research) collaboration projects aimed at improving healthcare for all children and adolescents in Europe, regardless of their background. The project results will be shared in various formats, including academic papers, lay reports, infographics, conference presentations, and social media videos. We will actively engage researchers, funder, and patient and clinical communities.
Let us know if you want to be involved or can connect us with relevant organizations!

Who is responsible for the project?

The Mi-CARE Priority Setting Partnership is coordinated and overseen by the **Mi-CARE Steering Group**: a group of 14 international healthcare professionals and people with lived experience of migration from diverse backgrounds and countries. The Steering Group is supported by a JLA advisor and an information specialist. More information about the Steering Group members can be found here: https://www.jla.nihr.ac.uk/priority-setting-partnerships/pediatric-migrant-health#tab-66961

Mi-CARE is a project of the Refugees and Migrants in Europe – Adolescent and Child Health (**REACH**) **network**, a Special Advisory Group of the **European Academy of Paediatrics (EAP** – www.eapaediatrics.eu/advisory-groups/reach/), composed of healthcare professionals actively involved in paediatric migrant healthcare in various countries of the European Region.

Mi-CARE is carried out in collaboration with the **James Lind Alliance,** overseen by the National Institute for Health and Care Research (NIHR – www.nihr.ac.uk), based at the NIHR Coordinating Centre (NIHRCC) at the University of Southampton.

For more information, please contact us at: micare.psp@gmail.com **or visit our website** www.jla.nihr.ac.uk/priority-setting-partnerships/pediatric-migrant-health.

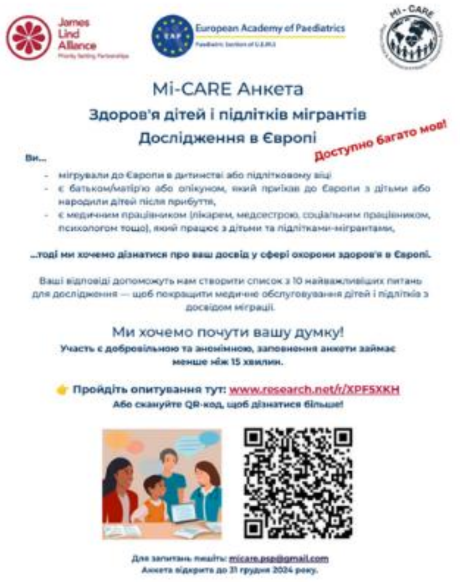

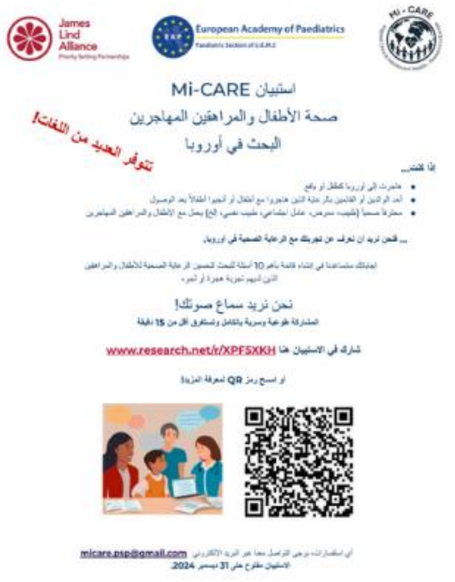

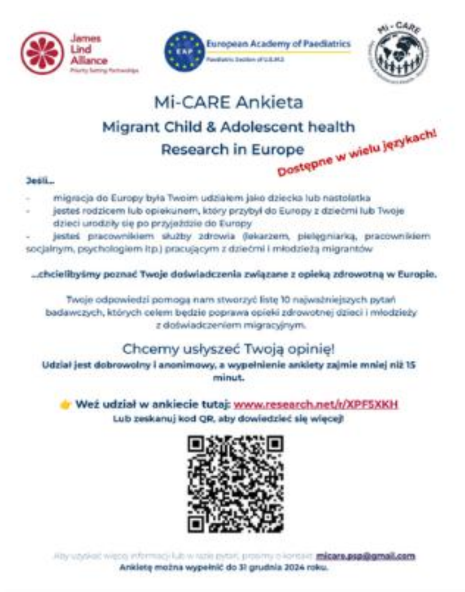

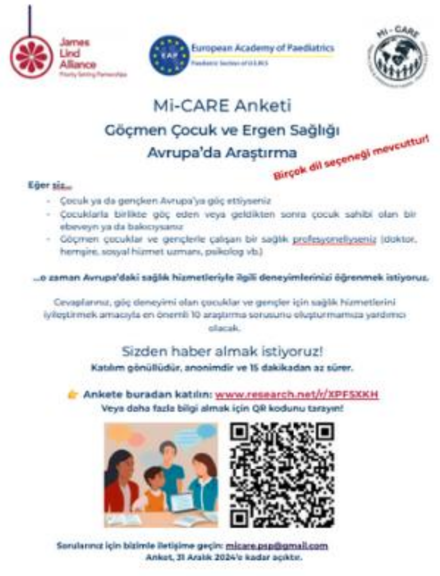

© 2026 Wiemker V et al. *JAMA Network Open*.

eMethods 3. First Mi-CARE consultation, October 14 to December 31, 2024

Note - this is the print out version of the survey. Online version was also available.

Mi-CARE Survey - ENGLISH

Migrant Child & Adolescent Health Research in Europe

We would like to hear about your experiences with healthcare in Europe. Your input will help us identify the most important questions for research to improve healthcare for children and teenagers with migration experiences. Participation is voluntary, anonymous, and takes about 15 minutes.

Thank you for taking our survey!

Survey Questions

While answering the following questions, imagine this situation: You have migrated to a new country, and either you or a child you care for becomes sick and needs medical attention. In these circumstances, there are many challenges you and the healthcare professionals might face…

1. What questions do you have concerned the healthcare of migrant children and teenagers in Europe?
Please enter your questions here. You are welcome to provide as many as you like.

____
____
____
____
____

2. What could be done to make healthcare better for children and teenagers who have moved to Europe?
Please enter your thoughts here. You are welcome to provide as many as you like.

____
____
____
____
____

About you

What describes you best?

- ☐ I arrived in Europe as a parent or carer with child / children / teenagers
- ☐ I had my child / children after arriving in Europe

© 2026 Wiemker V et al. *JAMA Network Open*.

- I came to Europe as a child / teenager with my parents or other adults
- I came to Europe as a child / teenager alone
- I am a healthcare worker (for example a nurse, doctor, psychologist, social worker)
- I am a healthcare worker with migration experience

Your Gender:

- Male
- Female
- Prefer not to say

How old are you?

Where are you from?

Where are you currently living?

What is your current migration status (if applicable)?

- Citizen of the country I migrated to
- Work visa
- Student visa
- Recognized refugee
- Asylum seeker
- Undocumented migrant

How old was your child / your children when you migrated (if applicable)? (if you migrated with several children, tick several boxes)

- Baby (<1 year)
- Young child (1 to 4 years)
- Schoolchild (5 to 12 years)
- Teenager (13 to 19 years)
- How old were you when you migrated to Europe?

What is your profession (answer only if you work in healthcare)?

- doctor
- nurse
- psychologist
- social worker
- Other (please specify):

In what area are you working (answer only if you work in healthcare)?

- Hospital
- Primary care
- Refugee arrival center

© 2026 Wiemker V et al. *JAMA Network Open*.

- ☐ Humanitarian Aid Organization
- ☐ NGO
- ☐ Other (please specify):

Anything else you want us to know?

Thank you for participating!

For more information about the Mi-CARE project, please contact us at: micare.psp@gmail.com; scan the QR code on the flyer or visit our website.

© 2026 Wiemker V et al. *JAMA Network Open*.

eMethods 4. Evidence Checking Framework, February 28 to March 10, 2025

Paediatric Migrant Health - Research in Europe

Evidence Checking

Thank you so much for your help with the Evidence Checking!

Objective

The **aim of** the **evidence-checking** process is **to systematically verify whether the research questions** identified through the Paediatric Migrant Health PSP **remain unanswered**. This ensures that priority research areas focus on genuine knowledge gaps and are based on both lived experiences and scientific evidence.

Only very high quality "evidence" is accepted to mark a question as "already sufficiently answered" and thus exclude it from the 2nd Mi-CARE Survey - so particularly in our under researched topic of paediatric migrant health, we do not expect that many SQ fall into this category. However, your work of doing a formal check of every single one is still really important!

In addition to being required before we can proceed to the next step of the Priority Setting Partnership, the Evidence Checking helps identify relevant literature in the field that is very useful for contextualizing the Mi-CARE results and Evidence Checking materials/results/documentation may even be the basis for a separate publication if you are interested :) Process Overview (according to the JLA Methodology)

Step 1: Defining the Research Scope

- Review the final list of summary research questions developed through PSP discussion.

- Ensure that questions meet the inclusion criteria:
    - They focus on paediatric migrant health in Europe.
    - They address healthcare issues specific to migrant children and adolescents.
    - They are formulated in a way that allows for empirical research.

Step 2: Identifying Existing Evidence

- Conduct systematic searches to determine whether each question has already been sufficiently addressed in existing research.
- Use the following prioritized evidence sources:
    - Systematic Review Databases (e.g., **Cochrane Library**)
    - Clinical Guidelines & Clinical Trials (e.g., WHO, ECDC, National Healthcare Institutions, via Google / Google Scholar)
    - PubMed (MEDLINE) for additional systematic reviews
- Exclusion Criteria:
    - Single, small-scale studies or country-specific reports that lack broad generalizability.
    - Research focusing solely on adult migrant health.

Step 3: Conducting the Literature Search

- When conducting your literature search, you may want to structure your search terms using the PICO scheme
- Recommended search parameters:

- **Timeframe**: Studies and reviews published after 2000, excluding older sources.
- **Language**: English-language publications, with the possibility of including other languages depending on available time and the language proficiency of the evidence checkers.
- **Population**: Studies focused on children (0-18 years) and, where relevant, young adults (19-24 years).
- Example for Search Terms: "migrant children", "migrant adolescents", "refugee children", "refugee adolescents", "migrant health", "pediatric migrant health", "Europe", "European countries"

Step 4: Categorizing the Research Questions/Documenting the classification

Each research question should be classified into one of the following categories:

1. **Answered:**
   - A high-quality systematic review, meta-analysis, or clinical guideline fully addresses the research question.
2. **Partially Answered:**
   - Some relevant systematic reviews or guidelines exist, but they do not comprehensively answer all aspects of the question.
   - Systematic reviews exist but are outdated (published before 2000).
   - Ongoing clinical trials suggest further research is still needed.
3. **Not Answered:**
   - No relevant systematic reviews, meta-analyses, or guidelines are found.
   - There is a clear evidence gap in the literature.
   - **O**nly small-size qualitative studies or opinion papers etc. exist or studies yield contradictory results

Use this spreadsheet to document your decision. You will find some examples for SQ searches and the corresponding documentation in the spreadsheet.

There may be minor changes to the summary questions in the coming days that we will immediately inform you about via Whatsapp. Please generously use the Whatsapp chat to bring up any issues that are unclear!

Step 5: Validation & Cross-Checking

The documentation of the evidence-checking process must follow a structured format to ensure transparency, traceability, and usability for future research publications (see the designated document for details).

Each classification should be clearly recorded, particularly for questions marked as **"answered"**, as these will receive special attention from Steering Group members for further review. While the Steering Group will not go through the entire evidence-checking process, they will focus on verifying whether the classification of certain questions as "answered" is well-supported by the documented evidence. If disagreements arise, an additional targeted search may be conducted, or subject-matter experts may be consulted.

Step 6: Documentation & Final Review

- Ensure that all **search processes, sources, and search terms** used for each question are systematically recorded.
- Document any **limitations** encountered, such as gaps in high-quality studies or regional disparities in research availability.
- Maintain **clear and structured documentation**.

© 2026 Wiemker V et al. *JAMA Network Open*.

eMethods 5. Second Mi-CARE consultation, March 20 to May 20, 2025

Note - this is the print out version of the survey. Online version was also available.

2nd Mi-CARE Survey
Your most Important Questions for Research

Share your perspective and help improve healthcare for children and adolescents with migration experience in Europe! Your input truly matters.
Taking part in this survey is completely voluntary and anonymous. We won't know who has filled it out. The survey will take about 5 to 15 minutes to complete.
Survey available in many languages! Ask for the language you need!

This is what you can do:
Here is a list of 56 questions about pediatric migrant health in Europe, categorized across seven key topics. These were developed from over 1,000 questions submitted during the first Mi-CARE Survey in 2024.

The topics are: **Language & Communication**, **Access to Care & Structural Barriers**, **Support Systems & Collaboration of different Actors**, **Health Disparities related to Migration**, **Vulnerable Groups / Special Needs**, **Mental Health & Trauma informed Care**, **Discrimination and Cultural Sensitivity**.

Now it's your turn to help decide which questions are the most important for future research!

Please choose the questions you find most important.

It is not necessary to choose questions from every theme. You may use the first (◌) column to preselect as many questions as you like (try not to select *all* questions). Use the second column (□) to **choose from your preselected questions up to 10 that should be the highest priority for research.**

Language & Communication

☐ What are the most effective ways to ensure **cost-free and easily accessible professional language support** for migrant children in healthcare institutions?
☐ What **impact** do **language barriers** have on the quality of care and health of migrant children?
☐ What are the **strengths and risks of different translation methods** in pediatric migrant healthcare (such as translation by family members, digital apps, online interpreters or volunteers)?
☐ Which **communication strategies** in healthcare settings are most effective in helping migrant children and their families explain their situations and understand medical information?
☐ How should **multimedia health information materials** (e.g. on common diseases, specific medical procedures, etc.) be designed to ensure accessibility for migrant families from diverse backgrounds?

Vulnerable Groups / Special Needs

☐ What impact does the **asylum process** have on the **physical and psychological health** of migrant children, and which measures can help reduce potential negative effects?
☐ What are effective trauma care approaches for migrant children who have undergone **female genital mutilation**?
☐ What are the effects of migration on **pregnancy outcomes** and the health and development of **unborn children**?
☐ What changes are needed to **reduce the fears of migrant children** without documents, such as concerns about deportation, so they feel safe accessing medical care?

© 2026 Wiemker V et al. *JAMA Network Open*.

☐ How can migrant children with **chronic illnesses, disabilities, and complex health needs** get timely and equitable access to healthcare (including medications, treatments, and assistive devices)?
☐ How can the **inappropriate use of medication** (e.g. prescription of sleep medication or tranquilizers instead of addressing trauma or living conditions) **be avoided** in treating refugee children and adolescents?
☐ What are alternative, effective strategies for **age determination** in adolescent refugees that avoid the use of [hand] X-rays?
☐ What approaches and tools are most effective for **recognizing and responding to signs of human trafficking** in children during medical screenings?
☐ What are the **specific healthcare needs and challenges** faced by **unaccompanied minor refugees**?

Mental Health & Trauma informed Care

☐ Which screening tools are most effective for **identifying mental health needs** in migrant children and adolescents?
☐ What can be done to reduce access barriers to mental healthcare for migrant children related to **stigma and limited knowledge about mental health**?
☐ What do migrant children and families envision as **essential elements of holistic pediatric healthcare**?
☐ What are the key factors in the healthcare system for creating **safe spaces** and building **trust** with migrant children and their families?
☐ What approaches of **trauma-informed care** can healthcare workers use to **minimize retraumatization** during medical care?
☐ What are the **most effective mental health programs** for addressing stress, anxiety, and trauma in migrant children and adolescents?
☐ What are effective **community-based mental health support** models for migrant children?

Discrimination & Cultural Sensitivity

☐ What **cultural competency training methods for healthcare workers** are most effective?
☐ In what ways does the **inclusion of healthcare workers from diverse backgrounds** impact the healthcare of migrant children?
☐ What impact do the **religious and cultural values** of migrant children have in medical consultations, for example regarding gender preferences?
☐ What **impact** do **racism and discrimination** have on the **quality of care** and (long-term) **health** of migrant children?
☐ What strategies can parents and healthcare workers use to **balance and integrate traditional cultural health practices with modern medical recommendations**?
☐ Is the **healthcare provided to migrant children different** from the healthcare provided to non-migrant children?
☐ What strategies effectively promote the **respectful and unbiased recognition of the autonomy and opinions** of migrant children and their families by healthcare workers?
☐ What approaches can be taken to provide **culturally sensitive sexual education and sexual healthcare** to migrant children and adolescents?
☐ What are the most effective approaches for integrating **cultural mediators with both medical and cultural expertise** into healthcare to address the specific needs of migrant children?

Support Systems & Collaboration of different Actors

☐ How can European healthcare systems **collaborate with other community support services** (e.g., social services, peer support) to provide more comprehensive care for migrant children?
☐ What structures are missing to ensure **continuity of medical care** for migrant children **across different countries**?
☐ What structures and processes are necessary to ensure **safe, accessible, and efficient sharing of migrant** children's **medical records** (such as vaccination history, previous illnesses, treatments, and test results)?
☐ What roles can **schools and community** programs play in ensuring access to **healthy nutrition, physical activity, and overall well-being** for migrant children?
☐ What strategies can improve **communication and coordination among different healthcare professionals, including specialists** (e.g. orthopedics or mental health)?

© 2026 Wiemker V et al. *JAMA Network Open*.

☐ How would a **standardized vaccination plan** across Europe impact immunization coverage for migrant children, especially those with unknown vaccination histories?
☐ What **financial and legal support** migrant children and their caregivers need to ensure access to medical care and guidance through health systems?
☐ How can healthcare services **involve parents and families more in supporting the health** of migrant children?

Health Disparities related to Migration

☐ How do social factors in the arrival country, such as **living conditions, education, and income**, affect the health of migrant children?
☐ Does **education about healthcare rights** improve health outcomes for migrant children?
☐ Are the **health issues faced by migrant children different** from the health issues faced by non-migrant children?
☐ In what ways does **migration impact the physical and mental health** of children?
☐ What **unique challenges** do only migrant children and their families encounter **in health emergencies**?
☐ What are the most effective **training approaches to help healthcare workers** understand the health effects of migration on children?
☐ What are the **differences in long-term health** between migrant and non-migrant children?

Access to Care & Structural Barriers

☐ What benefits does the **digitalization of healthcare processes** (such as insurance registration and booking appointments) offer for the care of migrant children?
☐ What are the best strategies to **provide clear and simple information about how the healthcare system works** in the country of arrival (including required documents, registration, insurance coverage, and access to medical care)?
☐ What are the **barriers** for migrant children and families to **access preventive medicine across Europe** (e.g. routine screenings and vaccinations)?
☐ How does the **complexity of navigating the healthcare system** (including registration and insurance) affect migrant childrens' access to care?
☐ What measures ensure that child healthcare services are **accessible in reception centers, asylum centers, and other temporary accommodations**?
☐ What **financial barriers** do migrant children and their families face in **healthcare emergencies**?
☐ What are the **main barriers** preventing migrant children, including those without documentation or health insurance, from **accessing healthcare**?
☐ What strategies or interventions can ensure healthcare services for **all migrant children** and teenagers (**universal access**)?

Why did you choose these questions as your top questions? Do you have any thoughts/comments you want to share? *(you do not have to fill in this field)*

Your role - please make sure to answer this question

What describes you best?

- I arrived in Europe as a parent or carer with child / children / teenagers
- I had my child / children after arriving in Europe
- I came to Europe as a child / teenager with my parents or other adults
- I came to Europe as a child / teenager alone
- I am a healthcare worker (for example a nurse, doctor, psychologist, social worker)
- I am a healthcare worker with migration experience

End of the Survey

© 2026 Wiemker V et al. *JAMA Network Open*.

Thank you so much for your participation in this survey!

If you know someone else whose experience would be valuable for our survey please ask them to participate. Every voice matters, and together we can make a difference.

If you are facing any issues or need help, please don't hesitate to contact us at **micare.psp@gmail.com**. While we are not able to handle immediate emergencies or solve every issue, we are here to listen and will do our best to help you find the right support or direct you to appropriate resources.

For more information about the **Mi-CARE** project, scan this QR code.

You can also access this survey online by scanning this QR code.

If you want to stay in touch with Mi-CARE and receive an email about the project results, write us an email at micare.psp@gmail.com.

Thank you for your contribution!

© 2026 Wiemker V et al. *JAMA Network Open*.

eMethods 6, Basel Final PSP Workshop Program

WORKSHOP AGENDA

June 4th: **Preparational Workshop**

Objective: Review of previous Mi-CARE project phases, finalizing of content & materials for work sessions with broader audience/community

Participants: Mi-CARE Workshop Core Team, Observers

TIME	SESSION	PRESENTER/ FACILITATOR	VENUE
**Until 09 00**	Arrival of PSP core team, *Breakfast* (Buffet opens at 6am)		Restaurant Hangar 9
**09 00 - 12 00**	**Core Team Session I**	Core Team	St. Exupery
**12 00 - 13 00**	*Lunch break*	Core Team	Montgolfiere Hotel Bar
**13 00 - 15 00**	**Core Team Session II**	Core Team	St. Exupery
**15 00 - 17 00**	Presentation of 2nd Mi-CARE Survey demographics & Finalization of materials	Veronika & Farah	St. Exupery
**17 00 - 18 00**	**Core Team Session III**	Core Team	St. Exupery
**19 00 - 21 00**	**Open for all workshop attendees:** shared dinner at **Markthallen Basel** (optional, expenses to be covered privately) https://altemarkthalle.ch/ *(leave together from the hotel lobby at 18 30)*		Markthallen AG Basel Steinentorberg 20 4051 Basel

© 2026 Wiemker V et al. *JAMA Network Open*.

June 5th: **Spotlights on Migrant Child Health in Europe**

Objective: Introduction to Mi-CARE project and workshop process, structured exchange on various aspects and national experiences of paediatric migrant health.

Participants: Core team, workshop participants and observers

TIME	SESSION	PRESENTER/ FACILITATOR	VENUE
**Until 09 00**	*Breakfast* (Buffet opens at 6am)		Restaurant Hangar 9
**09 00 - 09 30**	**Individual welcome and registration of all**	Loraine, Farah & Veronika	Welcome desk at reception
**09 30 - 10 15**	**Welcome & Get-to-Know Session**<br>Information on Workshop Videos / Pictures	Facilitated by Julia, & Vladimir	St. Exupery
**10 15 - 11 15**	**Orientation Session**<br>Information on Mi-CARE project and Agenda & Methodology of the Basel Workshop	Veronika & Farah<br>Bezawit<br>Toto	St. Exupery
**11 15 - 11 30**	*Coffee Break* (Collection of expectations for Final Priority Setting workshop)		
**11 30 - 12 30**	**Spotlight Session I:** Our Connections to Pediatric Migrant Health (see page 9)	Facilitated by Farah & Veronika	St. Exupery
**12 30 - 13 30**	*Lunch Break*		Montgolfiere Hotel Bar
**13 30 - 15 30**	**Spotlight Session II:** Our Connections to Pediatric Migrant Health	Facilitated by Farah & Veronika	St. Exupery
**15 30 - 16 00**	*Coffee Break*		
**16 00 - 16 45**	Group Discussion: **Why does (collaborative) research on pediatric migrant health matter?**	Facilitated by Julia	St. Exupery
**16 45 - 17 15**	Presentation of 2nd Mi-CARE Survey respondent demographics & debate	Farah & Veronika	St. Exupery
**17 15 - 17 30**	**"Why we care about Mi-CARE"** - Presentation of the short film	Vladimir	St. Exupery
**18 00 - 21 00**	Shared dinner at the **"Café zum goldige Velo"**, exploring Basel<br>https://www.human-gardening.ch/<br><br>*(leave together from hotel)*		Café zum goldige Velo<br>Muespacherstr, 76<br>4055 Basel

© 2026 Wiemker V et al. *JAMA Network Open*.

June 6th: **Formal Top 10 Priority Setting**

Objective: Structured process (adapted Nominal Group Technique) to identify and rank the final top 10 research priorities in pediatric migrant health.

Participants: Core team, workshop participants and observers

TIME	SESSION	PRESENTER/ FACILITATOR	VENUE
**Until 09 00**	*Breakfast* (Buffet opens at 6am)		Restaurant Hangar 9
**09 00 - 09 30**	*Arrival & Refreshments*		
**09 30 - 10 00**	**Opening Session**<br>Welcome and overview of the day<br>Keynote Input by Marcel Tanner, Swiss Tropical and Public Health Institute	Facilitated by Toto & Julia	St. Exupery
**10 00 - 11 10**	**Pre-workshop review exchange**	One JLA facilitator per small group (Menne, Mary-Rose, Toto)	St. Exupery, Petit Prince & Foyer
**11 10 - 11 40**	*Coffee Break*		
**11 40 - 12 45**	**First round of ranking**	One JLA facilitator per small group	St. Exupery, Petit Prince & Foyer
**12 45 - 13 45**	*Lunch break*		
**13 45 - 14 00**	**Review of progress so far**	Plenum – facilitated by JLA	St. Exupery
**14 00 - 15 10**	**Second round of ranking**	One JLA facilitator per small group	St. Exupery, Petit Prince & Foyer
**15 10 - 15 40**	*Coffee break*		
**15 40 - 16 00**	**Presenting the top 10 and combined ranking**	All participants	St. Exupery
**16 00 - 16 30**	**Close of formal workshop**<br>Information on delayed formal publication of priorities	Facilitated by Toto & Julia	St. Exupery
**17 00 - 21 00**	Celebration & Networking social evening with dinner at restaurant **“Pavillon im Park”**<br>https://parkpavillon.ch/<br>*(leave together at 16 30)*		Pavillon im Park<br>Schützenmattpark 1<br>4054 Basel

© 2026 Wiemker V et al. *JAMA Network Open*.

June 7th: **Dissemination Plan Development**

Objective: Prepare dissemination of the Mi-CARE project results, develop contextualization materials for Top 10 research priorities, co-develop a dissemination strategy, reflect on essential steps to promote meaningful migrant participation in research in future collaborations

Participants: Core team, selected workshop participants and observers

TIME	SESSION	PRESENTER/ FACILITATOR	VENUE
**Until 09 00**	*Breakfast* (Buffet opens at 6am)		Restaurant Hangar 9
**09 00 - 09 15**	Opening	Julia, Farah & Veronika	St. Exupery
**09 15 - 10 45**	**Development of Top 10 Research Priority Booklet** - Contextualization & Validation<br>Work in small groups & Hybrid Option	Toto, Nicole, Astrid, Valentina & Julia (Hybrid)	St. Exupery
**10 45 - 11 00**	*Coffee Break*		
**11 00 - 12 30**	**Dissemination & Publication**, “Getting migrant health research results to the people - how data and policy can come together”	Astrid & Susitha	St. Exupery
**12 30 - 13 30**	*Lunch Break*		
**13 30 - 15 30**	**Migrant Participation at the Core** - Identifying challenges and best practices of collaborative research	Nicole & Siobhán	St. Exupery
**15 30 - 17 00**	Finalization of the dissemination framework and closure of dissemination day	Farah & Veronika	St. Exupery
**Throughout the Day**	**Video Recording -** Mi-CARE Result Dissemination	Vladimir	St. Exupery,, Outside & Foyer
**19 00 - 21 00**	Social Event: **Rhine swim & Picknick** (optional, expenses to be covered privately)<br>https://www.basel.com/de/freizeit-ausfluege/rheinschwimmen<br>*(leave together at 18 30)*		

© 2026 Wiemker V et al. *JAMA Network Open*.

June 8th: **Consolidation of Results**

Objective: Consolidate workshop outcomes, document key insights, reflect on participant feedback and collaborative research process.

Participants: Core team, selected workshop participants and observers

TIME	SESSION	PRESENTER/ FACILITATOR	VENUE
**Until 09 00**	*Breakfast* (Buffet opens at 6am)		Restaurant Hangar 9
**09 00 - 10 30**	**Participant feedback collection**	Facilitated by Nicole	St. Exupery
**10 30 - 12 00**	**Core team observer analysis & reflections**	Observers; Facilitated by Nicole	St. Exupery
**12 00 - 13 00**	*Lunch Break*		
**13 00 - 15 00**	**Documentation of workshop results**	Facilitated by Farah	St. Exupery
**15 00 - 17 00**	**Final review and closing session**	Facilitated by Julia	St. Exupery

© 2026 Wiemker V et al. *JAMA Network Open*.

eTable 1. Key Definitions of Population Groups in the Study

Term	Definition	Notes / Source
**Migrant**	Person crossing an international border, regardless of documentation, duration, or reason for migration.	EU context: establishes or ceases usual residence in an EU/EFTA Member State for ≥12 months. Excludes tourists; may include seasonal/temporary workers. **Sources:** IOM Glossary on Migration[1]; UN Recommendations on Statistics of International Migration[2]; Regulation (EC) No 862/2007[3].
**Refugee**	Person granted refugee status or recognized protection by a host country due to a well-founded fear of persecution (race, religion, nationality, social group, political opinion).	Extended by the Cartagena Declaration (1984)[4] to include those fleeing generalised violence, conflict, or massive human rights violations. Protected under international law: 1951 Refugee Convention[5], 1967 Protocol[6]; UNHCR Guidelines[7].
**Asylum seeker**	Person seeking international protection in a country other than their own, awaiting a decision on refugee status.	If asylum is denied, the person may be required to leave the host country. **Sources:** 1951 Refugee Convention[5]; 1967 Protocol[6]; UNHCR Guidance[7].
**Child**	Person under 18 years of age.	UN Convention on the Rights of the Child[9].
**Adolescent**	Young person transitioning from childhood to adulthood	As per WHO definition[10], young people aged 10 to 19 years.
**Healthcare worker (HCW)**	Individual trained or certified to provide health services.	Includes physicians, nurses, midwives, allied health professionals (ie social worker, interpreter). When referring to health workers as study participants of this study, this term means health workers with relevant experience in treating migrant children on a regular base.
**Carer / Parent**	Adult providing primary care or guardianship for a child.	Includes biological/adoptive parents, legal guardians, or informal carers.
**Double expert**	HCW with professional and personal/family migration experience.	Offers dual perspective: clinical expertise and lived migration experience.

1. IOM. *Glossary on Migration*. 2019.
2. United Nations. *Recommendations on Statistics of International Migration*. 1998.
3. European Union. Regulation (EC) No 862/2007 on Community statistics on migration and international protection.
4. Cartagena Declaration on Refugees. 1984.
5. United Nations. *Convention Relating to the Status of Refugees*. 1951.
6. United Nations. *Protocol Relating to the Status of Refugees*. 1967.
7. UNHCR. *Guidelines on International Protection / Asylum Procedures*.
8. UNHCR. *Guiding Principles on Internal Displacement*. 1998.
9. United Nations. *Convention on the Rights of the Child*. 1989.
10. World Health Organisation. *Adolescent Health*.

© 2026 Wiemker V et al. *JAMA Network Open*.

eTable 2. Language Choices in the Mi-CARE Consultations

Consultation 1			
**Role**	**Used language (combined: video language, or survey language if no video language was chosen)**	**Count**	**Percent**
All roles	German	63	24.6
All roles	English	47	18.4
All roles	Ukrainian	28	10.9
All roles	Arabic	26	10.2
All roles	Italian	22	8.6
All roles	Farsi	20	7.8
All roles	Turkish	10	3.9
All roles	French	9	3.5
All roles	Somali	9	3.5
All roles	Amharic	8	3.1
All roles	Tigrinya	7	2.7
All roles	Spanish	4	1.6
All roles	Albanian	3	1.2
Double expert	English	11	33.3
Double expert	Arabic	5	15.2
Double expert	German	5	15.2
Double expert	Italian	4	12.1
Double expert	Farsi	2	6.1
Double expert	Somali	2	6.1
Double expert	Ukrainian	2	6.1
Double expert	Tigrinya	1	3.0
Double expert	Turkish	1	3.0
Healthworker	German	53	53.0
Healthworker	English	20	20.0
Healthworker	Italian	14	14.0
Healthworker	Ukrainian	8	8.0
Healthworker	French	4	4.0
Healthworker	Turkish	1	1.0
Migrant (child or parent/caregiver)	Arabic	21	17.1
Migrant (child or parent/caregiver)	Farsi	18	14.6
Migrant (child or parent/caregiver)	Ukrainian	18	14.6
Migrant (child or parent/caregiver)	English	16	13.0
Migrant (child or parent/caregiver)	Amharic	8	6.5
Migrant (child or parent/caregiver)	Turkish	8	6.5
Migrant (child or parent/caregiver)	Somali	7	5.7
Migrant (child or parent/caregiver)	Tigrinya	6	4.9
Migrant (child or parent/caregiver)	French	5	4.1

© 2026 Wiemker V et al. *JAMA Network Open*.

Migrant (child or parent/caregiver)	German	4	3.3
Migrant (child or parent/caregiver)	Italian	4	3.3
Migrant (child or parent/caregiver)	Spanish	4	3.3
Migrant (child or parent/caregiver)	Albanian	3	2.4

**Consultation 1**					
**Role**	**Used language**	**Video (Count)**	**Survey (Count)**	**Video (Percent)**	**Survey (Percent)**
All roles	Albanian	3	1	1.2	0.4
All roles	Amharic	8	2	3.1	0.8
All roles	Arabic	26	8	10.2	3.1
All roles	English	37	156	14.5	60.9
All roles	Farsi	20	3	7.8	1.2
All roles	French	9	6	3.5	2.3
All roles	German	54	48	21.1	18.8
All roles	I rather want to read about the survey in my language	21	NA	8.2	NA
All roles	Italian	22	16	8.6	6.2
All roles	Somali	9	NA	3.5	NA
All roles	Spanish	4	NA	1.6	NA
All roles	Tigrinya	7	NA	2.7	NA
All roles	Turkish	10	NA	3.9	NA
All roles	Ukrainian	25	16	9.8	6.2
All roles	NA	1	NA	0.4	NA
Double expert	Arabic	2	1	15.2	3.0
Double expert	English	9	26	27.3	78.8
Double expert	Farsi	2	2	6.1	6.1
Double expert	German	3	4	12.1	12.1
Double expert	I rather want to read about the survey in my language	1	4	9.1	12.1
Double expert	Italian	4	2	12.1	6.1
Double expert	Somali	2	1	6.1	3.0
Double expert	Tigrinya	1	1	3.0	3.0
Double expert	Turkish	1	1	3.0	3.0
Double expert	Ukrainian	2	2	6.1	6.1
Healthworker	English	16	46	16.0	46.0
Healthworker	French	4	4	4.0	4.0
Healthworker	German	45	36	45.0	36.0
Healthworker	I rather want to read about the survey in my language	1	12	1.0	12.0
Healthworker	Italian	14	9	14.0	9.0
Healthworker	Turkish	1	1	1.0	1.0
Healthworker	Ukrainian	8	7	8.0	7.0
Migrant (child or parent/caregiver)	Albanian	3	1	2.4	0.8
Migrant (child or parent/caregiver)	Amharic	8	2	6.5	1.6

© 2026 Wiemker V et al. *JAMA Network Open*.

Migrant (child or parent/caregiver)	Arabic	21	9	17.1	7.3
Migrant (child or parent/caregiver)	English	84	98	39.8	68.3
Migrant (child or parent/caregiver)	Farsi	18	14	14.6	9.8
Migrant (child or parent/caregiver)	French	5	6	4.1	4.1
Migrant (child or parent/caregiver)	German	48	7	21.8	4.1
Migrant (child or parent/caregiver)	I rather want to read about the survey in my language	4	3	3.3	3.3
Migrant (child or parent/caregiver)	Italian	4	5	3.3	4.1
Migrant (child or parent/caregiver)	Somali	7	3	3.3	2.4
Migrant (child or parent/caregiver)	Spanish	4	3	3.3	2.4
Migrant (child or parent/caregiver)	Tigrinya	6	4	4.9	3.3

**Consultation 2**			
**Role**	**Used language (combined: video language, or survey language if no video language was chosen)**	**Count**	**Percent**
All roles	German	171	29.7
All roles	English	153	26.6
All roles	French	100	17.4
All roles	Ukrainian	43	7.5
All roles	Arabic	24	4.2
All roles	Italian	20	3.5
All roles	Turkish	17	3.0
All roles	Spanish	11	1.9
All roles	Tigrinya	11	1.9
All roles	Albanian	8	1.4
All roles	Farsi	6	1.0
All roles	Amharic	5	0.9
All roles	Greek	3	0.5
All roles	Somali	3	0.5
All roles	Polish	1	0.2
Double expert	English	22	31.0
Double expert	German	13	18.3
Double expert	French	12	16.9
Double expert	Ukrainian	8	11.3
Double expert	Italian	5	7.0
Double expert	Arabic	3	4.2
Double expert	Turkish	3	4.2
Double expert	Farsi	2	2.8
Double expert	Spanish	2	2.8
Double expert	Albanian	1	1.4
Healthworker	German	144	39.8
Healthworker	English	104	28.7
Healthworker	French	78	21.5

Healthworker	Italian	14	3.9
Healthworker	Spanish	6	1.7
Healthworker	Ukrainian	5	1.4
Healthworker	Arabic	3	0.8
Healthworker	Greek	2	0.6
Healthworker	Turkish	2	0.6
Healthworker	Albanian	1	0.3
Healthworker	Amharic	1	0.3
Healthworker	Polish	1	0.3
Healthworker	Tigrinya	1	0.3
Migrant (child or parent/caregiver)	Ukrainian	30	21.0
Migrant (child or parent/caregiver)	English	27	18.9
Migrant (child or parent/caregiver)	Arabic	18	12.6
Migrant (child or parent/caregiver)	German	14	9.8
Migrant (child or parent/caregiver)	Turkish	12	8.4
Migrant (child or parent/caregiver)	French	10	7.0
Migrant (child or parent/caregiver)	Tigrinya	10	7.0
Migrant (child or parent/caregiver)	Albanian	6	4.2
Migrant (child or parent/caregiver)	Amharic	4	2.8
Migrant (child or parent/caregiver)	Farsi	3	2.1
Migrant (child or parent/caregiver)	Somali	3	2.1
Migrant (child or parent/caregiver)	Spanish	3	2.1
Migrant (child or parent/caregiver)	Greek	1	0.7
Migrant (child or parent/caregiver)	Italian	1	0.7

**Consultation 2**					
**Role**	**Used language**	**Video (Count)**	**Survey (Count)**	**Video (Percent)**	**Survey (Percent)**
Double expert	Albanian	2	2	2.8	2.8
Double expert	Arabic	8	17	11.3	23.9
Double expert	English	37	37	52.1	52.1
Double expert	Farsi	2	8	2.8	11.3
Double expert	French	10	2	14.1	2.8
Double expert	German	7	12	9.9	16.9
Double expert	Greek	NA	1	NA	1.4
Double expert	I rather want to read about the survey in my language	6	1	8.5	1.4

© 2026 Wiemker V et al. *JAMA Network Open*.

Double expert	I want to directly start with the Survey and don't...	23	NA	32.4	NA
Double expert	Italian	4	3	5.6	4.2
Double expert	Spanish	1	2	1.4	2.8
Double expert	Turkish	3	1	4.2	1.4
Double expert	Ukrainian	6	3	8.5	4.2
Healthworker	Albanian	1	1	0.3	0.3
Healthworker	Amharic	1	NA	0.3	NA
Healthworker	Arabic	3	3	0.8	0.8
Healthworker	English	49	127	13.5	35.1
Healthworker	Farsi	6	7	1.7	1.9
Healthworker	French	56	70	15.5	19.3
Healthworker	German	67	132	18.5	36.5
Healthworker	Greek	NA	9	NA	2.5
Healthworker	I rather want to read about the survey in my language	26	NA	7.2	NA
Healthworker	I want to directly start with the Survey and don't watch the video	136	NA	37.6	NA
Healthworker	Italian	11	6	3.0	1.7
Healthworker	Polish	NA	1	NA	0.3
Healthworker	Spanish	6	6	1.7	1.7
Healthworker	Tigrinya	1	NA	0.3	NA
Healthworker	Turkish	1	2	0.3	0.6
Healthworker	Ukrainian	3	5	0.8	1.4
Healthworker	NA	1	NA	0.3	NA
Migrant (child or parent/caregiver)	Albanian	3	6	2.1	4.2
Migrant (child or parent/caregiver)	Amharic	4	2	2.8	1.4
Migrant (child or parent/caregiver)	Arabic	16	15	11.2	10.5
Migrant (child or parent/caregiver)	English	11	46	7.7	32.2
Migrant (child or parent/caregiver)	Farsi	3	3	2.1	2.1
Migrant (child or parent/caregiver)	French	3	10	2.1	7.0
Migrant (child or parent/caregiver)	German	6	12	4.2	8.4
Migrant (child or parent/caregiver)	Greek	NA	1	NA	0.7
Migrant (child or parent/caregiver)	I rather want to read about the survey in my language	9	NA	6.3	NA
Migrant (child or parent/caregiver)	I want to directly start with the Survey and don't watch the video	36	NA	25.2	NA
Migrant (child or parent/caregiver)	Italian	1	1	0.7	0.7
Migrant (child or parent/caregiver)	Somali	2	3	1.4	2.1

© 2026 Wiemker V et al. *JAMA Network Open*.

Migrant (child or parent/caregiver)	Spanish	2	3	1.4	2.1
Migrant (child or parent/caregiver)	Tigrinya	7	8	4.9	5.6
Migrant (child or parent/caregiver)	Turkish	11	8	7.7	5.6
Migrant (child or parent/caregiver)	Ukrainian	27	25	18.9	17.5
Migrant (child or parent/caregiver)	NA	2	NA	1.4	NA
All roles	Albanian	5	7	0.9	1.2
All roles	Amharic	5	2	0.9	0.3
All roles	Arabic	21	20	3.6	3.5
All roles	English	68	210	11.8	36.5
All roles	Farsi	3	5	0.5	0.9
All roles	French	69	88	12.0	15.3
All roles	German	80	156	13.9	27.1
All roles	I rather want to read about the survey in my language	41	NA	7.1	NA
All roles	I want to directly start with the Survey and don't...	195	NA	33.9	NA
All roles	Italian	16	10	2.8	1.7
All roles	Somali	2	3	0.3	0.5
All roles	Spanish	9	11	1.6	1.9
All roles	Tigrinya	8	8	1.4	1.4
All roles	Turkish	15	11	2.6	1.9
All roles	Ukrainian	36	33	6.2	5.7
All roles	NA	3	NA	0.5	NA
All roles	Greek	NA	11	NA	1.9
All roles	Polish	NA	1	NA	0.2

© 2026 Wiemker V et al. *JAMA Network Open*.

eTable 3. List of the 53 Summary Questions

**Language and Communication**	
1.	What are the most effective ways to ensure cost-free and easily accessible professional language support for migrant children in healthcare institutions?
2.	What impact do language barriers have on the quality of care and health of migrant children?
3.	What are the strengths and risks of different translation methods in pediatric migrant healthcare, such as translation by family members, digital apps, online interpreters or volunteers,
4.	Which communication strategies in healthcare settings are most effective in helping migrant children and their families explain their situations and understand medical information?
5.	How should multimedia pediatric health information materials (e.g. on common diseases, specific medical procedures, etc.) be designed to ensure accessibility for migrant families from diverse backgrounds?
6.	What are the best strategies to provide clear and simple information about how the healthcare system works (including required documents, registration, insurance coverage, and how to get medical care)?
**Collaboration of different actors - Continuity of Care**	
7.	How can European healthcare systems collaborate with other community support services (e.g., social services, peer support) to provide more comprehensive care for migrant children?
8.	What roles can schools and community programs play in ensuring access to healthy nutrition, physical activity, and overall well-being?
9.	What structures are missing to ensure provide continuity of care across different countries?
10.	What strategies can improve communication and coordination among different healthcare professionals, including specialists (e.g. orthopaedics or mental health)?
11.	How would a standardized vaccination plan across Europe impact immunization coverage for migrant children, especially those with unknown vaccination histories?
12.	How can healthcare services involve parents and families more in supporting the health of migrant children?
**Vulnerable groups/Special needs**	
13.	What are the effects of migration on pregnancy outcomes and the health and development of unborn children?
14.	What approaches and tools are most effective for recognizing and responding to signs of human trafficking in children during medical screenings?
15.	What are the specific healthcare needs and barriers faced by unaccompanied minors?
16.	What are effective trauma care approaches for individuals who have undergone female genital mutilation?
17.	What changes are needed to reduce the fears of migrant children without documents, such as concerns about deportation, so they feel safe accessing medical care?
18.	What impact does the asylum and refugee process have on the physical and psychological health of migrant children and their families, and which measures can help reduce these effects?
19.	How can migrant children with chronic illnesses, disabilities, and complex health needs get timely and equitable access to healthcare (including medications, treatments, and assistive devices)?
20.	What are alternative, effective strategies for age determination in adolescent refugees that avoid the use of [hand] X-rays?
21.	How can the inappropriate use of medication (e.g. prescription of sleep medication or tranquilizers instead of addressing trauma or living conditions) be avoided in treating refugee children and adolescents?
**Mental health & Trauma Informed Care**	
22.	What can be done to reduce access barriers to mental healthcare related to stigma and limited knowledge about mental health?

© 2026 Wiemker V et al. *JAMA Network Open*.

23.	What are the key factors in the healthcare system for building safe spaces and trust with migrant families?
24.	What are effective community-based mental health support models for migrant children?
25.	What are the most effective mental health programs for addressing stress, anxiety, and trauma in migrant children and adolescents?
26.	What approaches of trauma-informed care can healthcare workers use to minimize retraumatization during medical care?
27.	Which screening tools are most effective for identifying mental health needs in migrant children and adolescents?
28.	What do migrant children and families envision as essential elements of holistic healthcare for migrant children?
**Access to care & Structural barriers**	
29.	What are the barriers for migrant children and families to access preventive medicine across Europe (e.g. routine screenings and vaccinations)?
30.	What financial barriers do migrant children and their families face in healthcare emergencies?
31.	How does the complexity of navigating the healthcare system (including registration and insurance) affect migrant children' s access to care?
32.	What strategies or interventions can ensure healthcare services for all migrant children and teenagers (universal access)?
33.	What are the main barriers preventing migrant children, including those without documentation or health insurance, from accessing healthcare?
34.	What measures ensure that healthcare services are accessible in reception centers, asylum centers, and other temporary accommodations?
35.	What financial and legal support migrant children and their caregivers need to ensure access to medical care and guidance through health systems?
36.	What structures and processes are necessary to ensure safe, accessible, and efficient sharing of migrant medical records (such as vaccination history, previous illnesses, treatments, and test results)?
37.	What benefits does the digitalization of healthcare processes (such as insurance registration and booking appointments) offer for the care of migrant children?
**Health disparities related to migration**	
38.	How do social factors in the arrival country, such as living conditions, education, and income, affect the health of migrant children?
39.	What are the differences in long-term health between migrant and non-migrant children?
40.	Are the health issues faced by migrant children different from the health issues faced by non-migrant children?
41.	In what ways does migration impact the physical and mental health of children?
42.	What are the most effective training approaches to help healthcare workers understand the health effects of migration on children?
43.	What unique challenges do only migrant children and their families encounter in health emergencies?
44.	Does education about healthcare rights improve health outcomes for migrant children?
**Discrimination & Cultural sensitivity**	
45.	What impact do racism and discrimination have on the quality of care and (long-term) health of migrant children?
46.	What strategies effectively promote the respectful and unbiased recognition of the autonomy and opinions of migrant children and their families by healthcare workers?
47.	What cultural competency training methods for healthcare workers are most effective?
48.	What strategies can parents and healthcare workers use to balance and integrate traditional cultural health practices with modern medical recommendations?
49.	What impact do the religious and cultural values of migrant families have in medical consultations, for example regarding gender preferences?

© 2026 Wiemker V et al. *JAMA Network Open*.

50.	What approaches can be taken to provide culturally sensitive sexual education and sexual healthcare to migrant children and adolescents?
51.	What are the most effective approaches for integrating cultural mediators with both medical and cultural expertise into healthcare to address the specific needs of migrant families?
52.	Is the healthcare provided to migrant children different from the healthcare provided to non-migrant children?
53.	In what ways does the inclusion of healthcare workers from diverse backgrounds impact the healthcare of migrant children?

© 2026 Wiemker V et al. *JAMA Network Open*.

eTable 4. Ranking Table by Respondent Group

Question	Top10_ALL	Top10_ALL_%	Rank_ALL	Important_ALL	Important_ALL_%	Top10_MIG	Top10_MIG_%	Rank_MIG	Important_MIG	Important_MIG_%	Top10_DEx	Top10_DEx_%	Rank_DEx	Important_DEx	Important_Dex_%	Top10_HW	Top10_HW_%	Rank_HW
MHT - What are the most effective mental health programs for addressing stress, anxiety, and trauma in migrant children and adolescents?	171	30%	1	291	51%	30	21%	8	57	40%	23	32%	2	37	52%	118	33%	1
DCS - What impact do racism and discrimination have on the quality of care and (long-term) health of migrant children?	161	28%	2	276	48%	42	29%	2	63	44%	23	32%	1	39	55%	96	27%	4
VGSN - How can migrant children with chronic illnesses, disabilities, and complex health needs get timely and equitable access to healthcare (including medications, treatments, and assistive devices)?	150	26%	3	263	46%	29	20%		51	36%	20	28%	5	33	46%	101	28%	3
LC - What are the most effective ways to ensure cost-free and easily accessible professional language support for migrant children in healthcare institutions?	144	25%	4	268	47%	28	20%		52	36%	13	18%		32	45%	103	28%	2
LC - What impact do language barriers have on the quality of care and health of migrant children?	144	25%	5	294	51%	45	31%	1	76	53%	21	30%	3	40	56%	78	22%	
VGSN - What impact does the asylum process have on the physical and psychological health of migrant children, and which measures can help reduce potential negative effects?	140	24%	6	262	45%	31	22%	7	53	37%	18	25%	8	35	49%	91	25%	6
HDM - In what ways does migration impact the physical and mental health of children?	138	24%	7	282	49%	28	20%		64	45%	17	24%		38	54%	93	26%	5
LC - Which communication strategies in healthcare settings are most effective in helping migrant children and their families explain their situations and understand medical information?	132	23%	8	248	43%	33	23%	3	55	38%	19	27%	6	30	42%	80	22%	10
ACSB - What strategies or interventions can ensure healthcare services for all migrant children and teenagers (universal access)?	125	22%	9	217	38%	28	20%		48	34%	14	20%		36	51%	83	23%	8
ACSB - What are the main barriers preventing migrant children, including those without documentation or health insurance, from accessing healthcare?	124	22%	10	243	42%	23	16%		49	34%	18	25%	9	29	41%	83	23%	7
VGSN - What are the specific healthcare needs and challenges faced by unaccompanied minor refugees?	122	21%		236	41%	23	16%		42	29%	17	24%		35	49%	82	23%	9
SSCA - What roles can schools and community programs play in ensuring access to healthy nutrition, physical activity, and overall well-being for migrant children?	118	20%		228	40%	30	21%	9	54	38%	20	28%	4	37	52%	68	19%	

© 2026 Wiemker V et al. *JAMA Network Open*.

DCS - What cultural competency training methods for healthcare workers are most effective?	111	19%		217	38%	24	17%		43	30%	13	18%		30	42%	74	20%	
HDM - How do social factors in the arrival country, such as living conditions, education, and income, affect the health of migrant children?	111	19%		226	39%	32	22%	4	54	38%	17	24%		32	45%	62	17%	
ACSB - What are the best strategies to provide clear and simple information about how the healthcare system works in the country of arrival (including required documents, registration, insurance coverage, and access to medical care)?	108	19%		214	37%	28	20%		43	30%	16	23%		30	42%	64	18%	
HDM - What are the most effective training approaches to help healthcare workers understand the health effects of migration on children?	106	18%		204	35%	20	14%		36	25%	16	23%		31	44%	70	19%	
MHT - Which screening tools are most effective for identifying mental health needs in migrant children and adolescents?	102	18%		215	37%	19	13%		36	25%	18	25%		34	48%	65	18%	
ACSB - What are the barriers for migrant children and families to access preventive medicine across Europe (e.g. routine screenings and vaccinations)?	102	18%		217	38%	30	21%	10	49	34%	15	21%		32	45%	57	16%	
VGSN - What changes are needed to reduce the fears of migrant children without documents, such as concerns about deportation, so they feel safe accessing medical care?	101	18%		217	38%	32	22%	5	50	35%	13	18%		27	38%	56	15%	
SSCA - How would a standardized vaccination plan across Europe impact immunization coverage for migrant children, especially those with unknown vaccination histories?	99	17%		193	34%	20	14%		36	25%	19	27%	7	29	41%	60	17%	
VGSN - What are effective trauma care approaches for migrant children who have undergone female genital mutilation?	97	17%		212	37%	15	10%		28	20%	14	20%		36	51%	68	19%	
HDM - What are the differences in long-term health between migrant and non-migrant children?	95	16%		200	35%	18	13%		45	31%	15	21%		34	48%	62	17%	
MHT - What approaches of trauma-informed care can healthcare workers use to minimize retraumatization during medical care?	94	16%		221	38%	13	9%		43	30%	12	17%		31	44%	69	19%	
MHT - What can be done to reduce access barriers to mental healthcare for migrant children related to stigma and limited knowledge about mental health?	94	16%		209	36%	29	20%		50	35%	10	14%		24	34%	55	15%	
VGSN - What are the effects of migration on pregnancy outcomes and the health and development of unborn children?	90	16%		188	33%	32	22%	6	47	33%	9	13%		31	44%	49	14%	
SSCA - What structures are missing to ensure continuity of medical care for migrant children across different countries?	88	15%		199	35%	13	9%		36	25%	14	20%		27	38%	61	17%	
SSCA - What structures and processes are necessary to ensure safe, accessible, and efficient sharing of migrant children's medical records (such as vaccination history, previous illnesses, treatments, and test results)?	87	15%		220	38%	17	12%		41	29%	13	18%		38	54%	57	16%	

© 2026 Wiemker V et al. *JAMA Network Open*.

DCS - What strategies can parents and healthcare workers use to balance and integrate traditional cultural health practices with modern medical recommendations?	87	15%		198	34%	21	15%		45	31%	18	25%	10	33	46%	48	13%	
HDM - What unique challenges do only migrant children and their families encounter in health emergencies?	82	14%		202	35%	20	14%		47	33%	15	21%		36	51%	47	13%	
HDM - Are the health issues faced by migrant children different from the health issues faced by non-migrant children?	82	14%		187	32%	25	17%		49	34%	15	21%		30	42%	42	12%	
VGSN - What approaches and tools are most effective for recognizing and responding to signs of human trafficking in children during medical screenings?	81	14%		179	31%	16	11%		34	24%	13	18%		27	38%	52	14%	
DCS - What approaches can be taken to provide culturally sensitive sexual education and sexual healthcare to migrant children and adolescents?	81	14%		177	31%	19	13%		34	24%	16	23%		32	45%	46	13%	
ACSB - What measures ensure that child healthcare services are accessible in reception centers, asylum centers, and other temporary accommodations?	80	14%		191	33%	16	11%		36	25%	12	17%		27	38%	52	14%	
LC - What are the strengths and risks of different translation methods in pediatric migrant healthcare (such as translation by family members, digital apps, online interpreters or volunteers)?	80	14%		186	32%	26	18%		45	31%	12	17%		28	39%	42	12%	
MHT - What are the key factors in the healthcare system for creating safe spaces and building trust with migrant children and their families?	79	14%		193	34%	19	13%		47	33%	13	18%		31	44%	47	13%	
SSCA - How can European healthcare systems collaborate with other community support services (e.g., social services, peer support) to provide more comprehensive care for migrant children?	77	13%		195	34%	18	13%		41	29%	8	11%		30	42%	51	14%	
SSCA - What financial and legal support migrant children and their caregivers need to ensure access to medical care and guidance through health systems?	75	13%		187	32%	22	15%		49	34%	9	13%		28	39%	44	12%	
ACSB - How does the complexity of navigating the healthcare system (including registration and insurance) affect migrant childrens' access to care?	73	13%		189	33%	20	14%		41	29%	9	13%		24	34%	44	12%	
DCS - Is the healthcare provided to migrant children different from the healthcare provided to non-migrant children?	73	13%		203	35%	19	13%		51	36%	11	15%		27	38%	43	12%	
DCS - What impact do the religious and cultural values of migrant children have in medical consultations, for example regarding gender preferences?	71	12%		160	28%	16	11%		33	23%	13	18%		29	41%	42	12%	
SSCA - What strategies can improve communication and coordination among different healthcare professionals, including specialists (e.g. orthopedics or mental health)?	71	12%		182	32%	25	17%		47	33%	10	14%		26	37%	36	10%	
VGSN - How can the inappropriate use of medication (e.g. prescription of sleep medication or tranquilizers instead of addressing trauma or living conditions) be avoided in treating refugee children and adolescents?	66	11%		164	28%	17	12%		45	31%	9	13%		22	31%	40	11%	

© 2026 Wiemker V et al. *JAMA Network Open*.

DCS - What are the most effective approaches for integrating cultural mediators with both medical and cultural expertise into healthcare to address the specific needs of migrant children?	65	11%		169	29%	14	10%		36	25%	11	15%		26	37%	40	11%	
SSCA - How can healthcare services involve parents and families more in supporting the health of migrant children?	65	11%		178	31%	21	15%		39	27%	11	15%		26	37%	33	9%	
ACSB - What financial barriers do migrant children and their families face in healthcare emergencies?	65	11%		163	28%	24	17%		50	35%	12	17%		25	35%	29	8%	
LC - How should multimedia health information materials (e.g. on common diseases, specific medical procedures, etc.) be designed to ensure accessibility for migrant families from diverse backgrounds?	63	11%		155	27%	21	15%		37	26%	9	13%		23	32%	33	9%	
MHT - What are effective community-based mental health support models for migrant children?	62	11%		177	31%	23	16%		48	34%	10	14%		24	34%	29	8%	
MHT - What do migrant children and families envision as essential elements of holistic pediatric healthcare?	57	10%		143	25%	14	10%		32	22%	10	14%		22	31%	33	9%	
DCS - In what ways does the inclusion of healthcare workers from diverse backgrounds impact the healthcare of migrant children?	57	10%		140	24%	14	10%		35	24%	11	15%		27	38%	32	9%	
DCS - What strategies effectively promote the respectful and unbiased recognition of the autonomy and opinions of migrant children and their families by healthcare workers?	53	9%		149	26%	13	9%		28	20%	10	14%		22	31%	30	8%	
ACSB - What benefits does the digitalization of healthcare processes (such as insurance registration and booking appointments) offer for the care of migrant children?	43	7%		106	18%	15	10%		26	18%	8	11%		17	24%	20	6%	
HDM - Does education about healthcare rights improve health outcomes for migrant children?	43	7%		128	22%	13	9%		33	23%	12	17%		27	38%	18	5%	
VGSN - What are alternative, effective strategies for age determination in adolescent refugees that avoid the use of [hand] X-rays?	35	6%		92	16%	6	4%		21	15%	4	6%		18	25%	25	7%	

Legenda

LC = Language & Communication. SSCA = Collaboration of different actors - continuity of care. VGSN = Vulnerable groups/Special needs. MHT = Mental health & Trauma Informed Care. ACSB = Access to care & structural barriers. HDM = Health disparities related to migration. DCS = Discrimination & Cultural sensitivity

ALL = All respondents. MIG = Migrant respondents. Dex = Double expert respondents. HW = Healthworker respondents.

© 2026 Wiemker V et al. *JAMA Network Open*.

eTable 5. Top 25 Research Priorities on Pediatric Migrant Health in Europe, Based on the First and Second Mi-CARE Consultations (Random Order of Presentation)

1. What impact does the asylum process have on the physical and psychological health of migrant children, and which measures can help reduce potential negative effects?
2. In what ways does migration impact the physical and mental health of children?
3. How do social factors in the arrival country, such as living conditions, education, and income, affect the health of migrant children?
4. What are the best strategies to provide clear and simple information about how the healthcare system works in the country of arrival (including required documents, registration, insurance coverage, and access to medical care)?
5. What strategies can parents and healthcare workers use to balance and integrate traditional cultural health practices with modern medical recommendations?
6. How can healthcare services involve parents and families more in supporting the health of migrant children?
7. What are the specific healthcare needs and challenges faced by unaccompanied minor refugees?
8. How would a standardized vaccination plan across Europe impact immunization coverage for migrant children, especially those with unknown vaccination histories?
9. Which communication strategies in healthcare settings are most effective in helping migrant children and their families explain their situations and understand medical information?
10. How can migrant children with chronic illnesses, disabilities, and complex health needs get timely and equitable access to healthcare (including medications, treatments, and assistive devices)?
11. What roles can schools and community programs play in ensuring access to healthy nutrition, physical activity, and overall well-being for migrant children?
12. What can be done to reduce access barriers to mental healthcare for migrant children related to stigma and limited knowledge about mental health?
13. What are the most effective mental health programs for addressing stress, anxiety, and trauma in migrant children and adolescents?
14. What approaches of trauma-informed care can healthcare workers use to minimize retraumatization during medical care?
15. What are the barriers for migrant children and families to access preventive medicine across Europe (e.g. routine screenings and vaccinations)?
16. What impact do language barriers have on the quality of care and health of migrant children?
17. What are the most effective training approaches to help healthcare workers understand the health effects of migration on children?
18. Which screening tools are most effective for identifying mental health needs in migrant children and adolescents?
19. What changes are needed to reduce the fears of migrant children without documents, such as concerns about deportation, so they feel safe accessing medical care?
20. What impact do racism and discrimination have on the quality of care and (long-term) health of migrant children?
21. What are the most effective ways to ensure cost-free and easily accessible professional language support for migrant children in healthcare institutions?

© 2026 Wiemker V et al. *JAMA Network Open*.

22.	What cultural competency training methods for healthcare workers are most effective?
23.	What are the main barriers preventing migrant children, including those without documentation or health insurance, from accessing healthcare?
24.	What are the effects of migration on pregnancy outcomes and the health and development of unborn children?
25.	What strategies or interventions can ensure healthcare services for all migrant children and teenagers (universal access)?

© 2026 Wiemker V et al. *JAMA Network Open*.

eTable 6. Mi-CARE Final Workshop Participants

Role	First Name	Last Name	Gender	Country of Residence	Country of Origin
double experts					
double expert 1	Mariia	Teslenko	Female	Poland	Ukraine
double expert 2	Hiba	Akyol	Female	Germany	Turkey
double expert 3	Olena	Nyankovska	Female	Poland	Ukraine
double expert 4	Toktam	Pour	Female	Germany	Iran
double expert 5	Earta	Lushaku	Female	Germany	Kosovo
double expert 6	Kolahta	Ioab	Female	Germany	Erithrea
double expert 7	Zeynep Muradiye	Ünsal	Female	Germany	Turkey
double expert 8	Alla	Babekr	Female	Germany	Sudan
migrants					
migrant 1	Bezawit	Sima	Female	Norway	Ethiopia
migrant 2	Michael	Asonganyi	Male	Norway	Kamerun
migrant 3	Afona	Chernet	Male	Switzerland	Eritrea
migrant 4	Zabihullah	Khrosh	Male	Italy	Afghanistan
migrant 5	Yaatsil	Gonzalez	Female	Germany	Mexico
migrant 6	Grace Yuying	Gao	Female	Switzerland	China
migrant 7	Fatma	Sahindal	Female	Switzerland	Turkey
health workers					
health worker 1	Agapios	Terzidis	Male	Greece	Not disclosed
health worker 2	Siobhán	Neville	Female	Ireland	Not disclosed
health worker 3	Lindsay	Osei	Male	France	Not disclosed
health worker 4	Amy	Stevens	Female	UK	Not disclosed
health worker 5	Valentina	Burzio	Female	Italy	Not disclosed
health worker 6	Alexandra	Kruse	Fe.male	Denmark	Not disclosed

© 2026 Wiemker V et al. *JAMA Network Open*.

eTable 7. Mi-CARE Final Workshop: Combined Group's Ranking of Top 25 Priorities (Arithmetic and Geometric Mean)[1]

Rank	Question	Group 1	Group 2	Group 3	Arith-metic mean rank	Geo-metric mean rank
**1**	What strategies or interventions can ensure healthcare services for all migrant children and teenagers (**universal access**)?	1	1	1	1.0	**1.0**
**2**	What impact do **racism and discrimination** have on the quality of care and (long-term) health of migrant children?	4	2	3	3.0	**2.9**
**3**	What are the **main barriers** preventing migrant children, including those without documentation or health insurance, from **accessing healthcare**?	2	3	9	4.7	**3.8**
**4**	In what ways does **migration impact the physical and mental health** of children?	18	5	2	8.3	**5.6**
**5**	How can migrant children with **chronic illnesses, disabilities, and complex health needs** get timely and equitable access to healthcare (including medications, treatments, and assistive devices)?	9	4	7	6.7	**6.3**
**6**	How do **social factors in the arrival country**, such as living conditions, education, and income, affect the health of migrant children?	10	8	4	7.3	**6.8**
**7**	What are the most effective **training** approaches to help healthcare workers understand the **health effects of migration** on children?	5	6	12	7.7	**7.1**
**8**	What are the specific healthcare needs and challenges faced by **unaccompanied minor refugees**?	7	9	8	8.0	**8.0**
**9**	What are the most effective ways to ensure **cost-free and easily accessible professional language support** for migrant children in healthcare institutions?	3	14	14	10.3	**8.4**
**10**	How can healthcare services **involve parents and families** more in supporting the health of migrant children?	6	10	13	9.7	**9.2**
**11**	What **impact** does the **asylum process** have on the physical and psychological health of migrant children, and which measures can help reduce potential negative effects?	14	12	5	10.3	**9.4**
**12**	What **roles can schools and community programs** play in ensuring access to healthy nutrition, physical activity, and overall well-being for migrant children?	13	11	6	10.0	**9.5**
**13**	What are the most effective **mental health programs** for addressing **stress, anxiety, and trauma** in migrant children and adolescents?	8	18	11	12.3	**11.7**
**14**	What are the best strategies to provide clear and simple **information about how the healthcare system works** in the country of arrival (including required documents, registration, insurance coverage, and access to medical care)?	16	7	18	13.7	**12.6**

[1]The geometric mean was used as a more robust measure method of rank aggregation for scores involving skewed data. High ranking in any group is directly reflected in the combined ranking by geometric mean. The geometric mean is more respectful of the differences in the three groups then an arithmetic mean.
For each priority, the geometric mean rank was calculated by multiplying the ranks assigned by each group and taking the n-th root (where n equals the number of groups).

**15**	Which **communication strategies** in healthcare settings are most effective in helping migrant children and their families explain their situations and understand medical information?	17	13	10	13.3	**13.0**
**16**	What are the effects of migration on **pregnancy outcomes** and the **health and development of unborn children**?	12	15	15	14.0	**13.9**
**17**	How would a **standardized vaccination** plan across Europe impact immunization coverage for migrant children, especially those with unknown vaccination histories?	11	16	17	14.7	**14.4**
**18**	What **cultural competency training** methods for healthcare workers are most effective?	15	17	16	16.0	**16.0**
**19**	What approaches of trauma-informed care can healthcare workers use to **minimize retraumatization during medical care**?	19	19	19	19.0	**19.0**
**20**	What **impact** do **language barriers** have on the quality of care and health of migrant children?	20	20	20	20.0	**20.0**
**21**	What changes are needed to reduce the **fears of migrant children without documents**, such as concerns about deportation, so they feel safe accessing medical care?	21	21	21	21.0	**21.0**
**22**	Which **screening tools** are most effective for identifying mental health needs in migrant children and adolescents?	24	22	22	22.7	**22.6**
**23**	What can be done to **reduce access barriers to mental healthcare** for migrant children related to stigma and limited knowledge about mental health?	22	23	23	22.7	**22.7**
**24**	What strategies can parents and healthcare workers use to **balance and integrate traditional cultural health practices** with modern medical recommendations?	23	25	25	24.3	**24.3**
**25**	What are the **barriers** for migrant children and families **to access preventive medicine** across Europe (e.g. routine screenings and vaccinations)?	25	24	24	24.3	**24.3**

© 2026 Wiemker V et al. *JAMA Network Open*.

eFigure 1. Language Distributions in the First and Second Mi-CARE Consultations by Respondents' Role

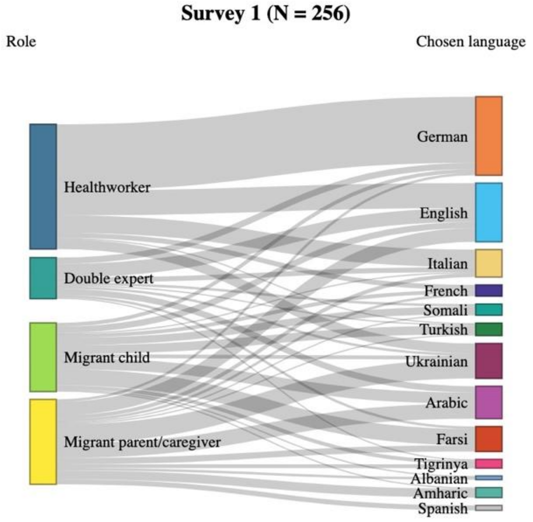

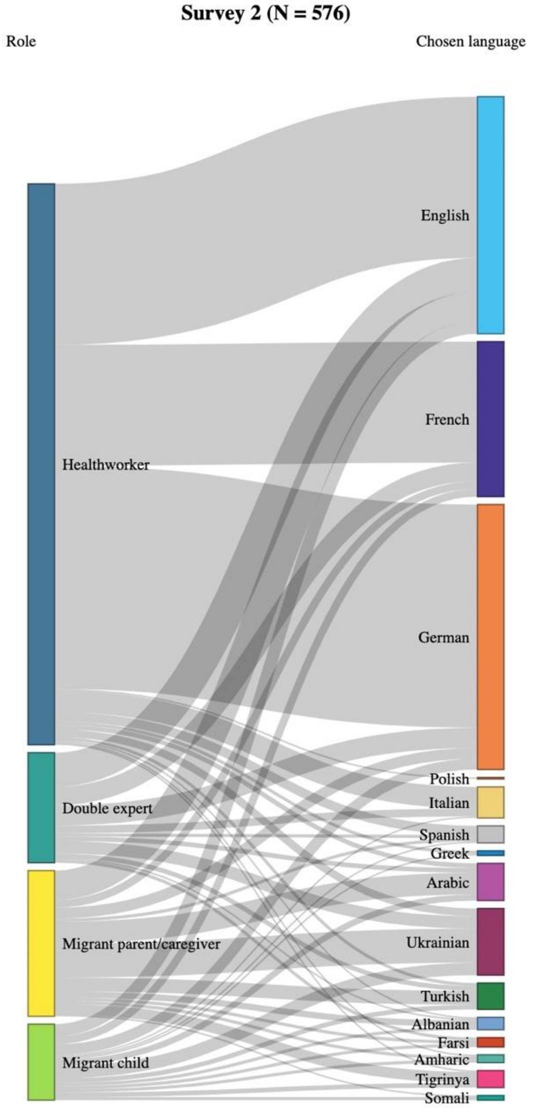

© 2026 Wiemker V et al. *JAMA Network Open*.

eFigure 2. Strategic Research Framework of Identified Priorities

Following identification of the top 10 research priorities, an operational strategic framework visualizing their interrelatedness wasdeveloped during the consensus workshop and further elaborated by the REACH group.

Research on strategies, models, and interventions to achieve universal health access for migrant children and young people (priority 1) represent the overarching priority, aligned with WHO guidance, such as the WHO global action plan on promoting the health of refugees and migrants 2019–2030. This goal is supported by evidence on effective structural and social support systems for pediatric migrants (priorities 1, 2, 3, 5, 6, 9) and on communication, partnership, and mutual learning between migrant families and health workers (priorities 7, 10), informed by linked migration data. All these components are grounded in an understanding of the mechanisms of physical, mental, and social wellbeing of the child across its migration journey (priorities 2, 4, 6, 8).

Grounded in the lived and professional experience of those directly concerned, the framework is intended to be refined iteratively as it is applied across diverse health-system and migration contexts. As an operational model, it may guide researchers and funders in mapping and choosing research questions, funding priorities, and evaluation strategies to improve health across the migration trajectory.

© 2026 Wiemker V et al. *JAMA Network Open*.

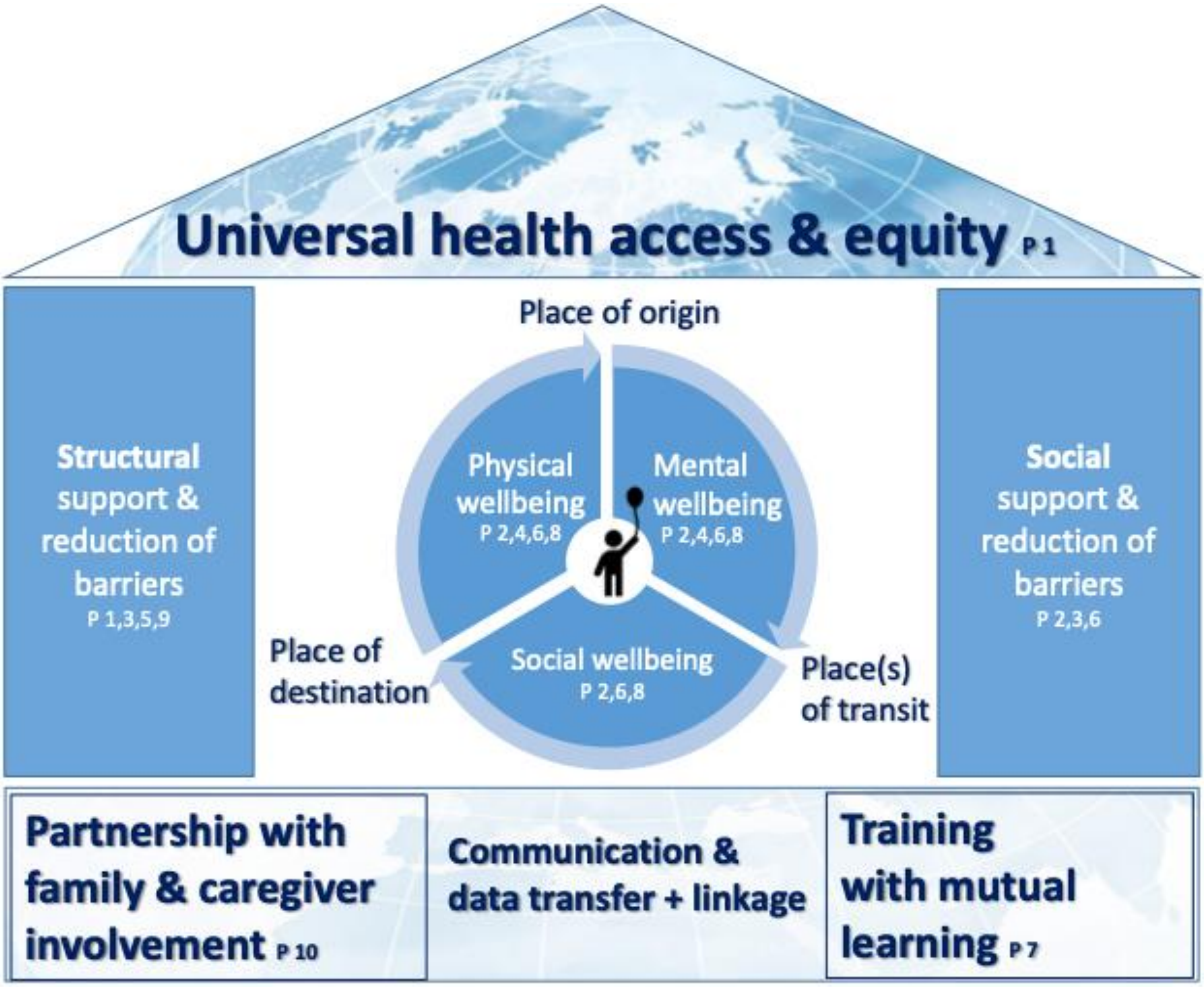

P	Top 10 research priorities		
1	Strategies to ensure universal access	6	Influence of socio-economic factors in host countries
2	Impact of racism and discrimination	7	Training for healthcare workers
3	Barriers to accessing healthcare	8	Specific healthcare needs of unaccompanied minors
4	Impact of migration on physical and mental health	9	Access to professional language support
5	Chronic and complex conditions	10	Strengthening parental involvement

© 2026 Wiemker V et al. *JAMA Network Open*.
