# Supplementary material for: Partnering With Caregivers and Clinicians to Determine Research Priorities in Pediatric Migrant Health
Source: JAMA Netw Open. 2026 Jul 29;9(7):e2626087. doi: 10.1001/jamanetworkopen.2026.26087 (PMC13421195; doi:10.1001/jamanetworkopen.2026.26087)
Supplement: Supplement 2. — Nonauthor Collaborators [file jamanetwopen-e2626087-s002.pdf]

Supplemental Online Content: Nonauthor Collaborators
*First name, last name, and suffix (if applicable) are required and will appear in PubMed.

| *Group Name(s): Refugees and Migrants in Europe—Adolescent and Child Health (REACH) network | | | | |
|---|---|---|---|---|
| ***First Name and Middle Initial(s)** | ***Last Name** | ***Suffix (eg, Jr, III)** | Location (city, state/province, country) | Role or Contribution, eg, chair, principal investigator |
| Ruud | Nijman | | United Kingdom | Co-Chair |
| Ghena | Alhadwah | | Syria | Member |
| Saleh | Aljadeeah | | Belgium | Member |
| Marta | Alustiza | | Germany | Member |
| Albertine | Baauw | | The Netherlands | Member |
| Francesca | Bisio | | France | Member |
| Sina | Buser | | Germany | Member |
| Liselot | De Cloedt | | Belgium | Member |
| Ann | De Guchtenaere | | Belgien | Member |
| Sarah | Eisen | | United Kingdom | Member |
| Nese | Gadzama | | Ireland | Member |
| Juliette | Goutines | | France | Member |
| Yaatsil | Guevara Gonzalez | | Mexico/Germany | Member |
| Kolahta | Ioab | | Germany | Member |
| Evika | Karamagioli | | Greece | Member |
| Nora | Karara | | Germany | Member |
| Alexandra | Kruse | | United Kingdom | Member |
| Martina | Lembani | | South Africa | Member |
| Marina | Mamenko | | Ukraine | Member |
| Artur | Mazur | | Poland | Member |
| Sonia | Milkova | | Spain | Member |
| Nadja | Naef | | Switzerland | Member |
| Siobhan | Neville | | Ireland | Member |
| Olena | Nyankovska | | Poland | Member |
| Manuela | Orjuela | | Germany | Member |
| Lindsay | Osei | | France | Member |

Supplemental Online Content: Nonauthor Collaborators
*First name, last name, and suffix (if applicable) are required and will appear in PubMed.

| ***First Name and Middle Initial(s)** | ***Last Name** | ***Suffix (eg, Jr, III)** | Location (city, state/province, country) | Role or Contribution, eg, chair, principal investigator |
|---|---|---|---|---|
| Berrak | Öztosun | | Turkey | Member |
| Nora | Poey | | France | Member |
| Toktam | Pour | | Germany | Member |
| Aneta | Radaczyńska | | Poland | Member |
| Margherita | Rosa | | Italy | Member |
| Talia | Sainz | | Spain | Member |
| Farhan | Saleem Ud Din | | Norway | Member |
| Nuria | Sanchez Clemente | | United Kingdom | Member |
| Francesca | Seregni | | United Kingdom | Member |
| Judith | Söller | | Austria | Member |
| Amy | Stevens | | United Kingdom | Member |
| Agis | Terzidis | | Greece | Member |
| Paul | Torpiano | | Malta/UK | Member |
| Eviana | Tsantzali | | Greece | Member |
| Ulrich | von Both | | Germany | Member |
| Antonia | Walther | | Germany | Member |
| Anko | Wolting | | Netherlands | Member |
