# Supplementary material for: Partnering With Caregivers and Clinicians to Determine Research Priorities in Pediatric Migrant Health
Source: JAMA Netw Open. 2026 Jul 29;9(7):e2626087. doi: 10.1001/jamanetworkopen.2026.26087 (PMC13421195; doi:10.1001/jamanetworkopen.2026.26087)
Supplement: Supplement 3. — Data Sharing Statement [file jamanetwopen-e2626087-s003.pdf]

Data Sharing Statement

Data
Data available: Yes
Data types: Deidentified participant data
How to access data: data will be made available upon request: julia.brandenberger@insel.ch
When available: With publication

Supporting Documents
Document types: None

Additional Information
Who can access the data: researchers whose proposed use of the data has been approved
Types of analyses: for research
Mechanisms of data availability: after approval of a proposal
